# Characterization of lysosomal proteins Progranulin and Prosaposin and their interactions in Alzheimer’s disease and aged brains: increased levels correlate with neuropathology

**DOI:** 10.1186/s40478-019-0862-8

**Published:** 2019-12-21

**Authors:** Anarmaa Mendsaikhan, Ikuo Tooyama, Jean-Pierre Bellier, Geidy E. Serrano, Lucia I. Sue, Lih-Fen Lue, Thomas G. Beach, Douglas G. Walker

**Affiliations:** 1 Molecular Neuroscience Research Center, Shiga University of Medical Science, Seta, Otsu, Otsu, 520-2192 Japan; 2 Civin Neuropathology Laboratory, Banner Sun Health Research Institute, Sun City, AZ USA; 3 School of Life Sciences and Neurodegenerative Disease Research Center, Biodesign Institute, Arizona State University, Tempe, AZ USA

**Keywords:** Amyloid, Aggregation, Growth factors, Alzheimer’s disease, Progranulin, Prosaposin, Neuropathology, Tangles, Interactions

## Abstract

Progranulin (PGRN) is a protein encoded by the GRN gene with multiple identified functions including as a neurotrophic factor, tumorigenic growth factor, anti-inflammatory cytokine and regulator of lysosomal function. A single mutation in the human GRN gene resulting in reduced PGRN expression causes types of frontotemporal lobar degeneration resulting in frontotemporal dementia. Prosaposin (PSAP) is also a multifunctional neuroprotective secreted protein and regulator of lysosomal function. Interactions of PGRN and PSAP affect their functional properties. Their roles in Alzheimer’s disease (AD), the leading cause of dementia, have not been defined. In this report, we examined in detail the cellular expression of PGRN in middle temporal gyrus samples of a series of human brain cases (*n* = 45) staged for increasing plaque pathology. Immunohistochemistry showed PGRN expression in cortical neurons, microglia, cerebral vessels and amyloid beta (Aβ) plaques, while PSAP expression was mainly detected in neurons and Aβ plaques, and to a limited extent in astrocytes. We showed that there were increased levels of PGRN protein in AD cases and corresponding increased levels of PSAP. Levels of PGRN and PSAP protein positively correlated with amyloid beta (Aβ), with PGRN levels correlating with phosphorylated tau (serine 205) levels in these samples. Although PGRN colocalized with lysosomal-associated membrane protein-1 in neurons, most PGRN associated with Aβ plaques did not. Aβ plaques with PGRN and PSAP deposits were identified in the low plaque non-demented cases suggesting this was an early event in plaque formation. We did not observe PGRN-positive neurofibrillary tangles. Co-immunoprecipitation studies of PGRN from brain samples identified only PSAP associated with PGRN, not sortilin or other known PGRN-binding proteins, under conditions used. Most PGRN associated with Aβ plaques were immunoreactive for PSAP showing a high degree of colocalization of these proteins that did not change between disease groups. As PGRN supplementation has been considered as a therapeutic approach for AD, the possible involvement of PGRN and PSAP interactions in AD pathology needs to be further considered.

## Introduction

Alzheimer’s disease (AD) is the most frequent cause of dementia in elderly populations and currently affecting an estimated 47 million people worldwide [1], but as the ages of populations in most countries are increasing, the incidence of AD will significantly increase. The brains of AD-affected subjects show accumulations of amyloid beta (Aβ) plaques and neurofibrillary tangles (NFT), the hallmark pathological features of this disease [2]. Preventing the formation of these pathological structures is considered the key to preventing cognitive decline, the main clinical feature of AD, but the mechanisms and sequence of events leading to the accumulation of plaques and tangles, neuronal death and cognitive decline are not fully understood. To date, in spite of promising experimental data in AD animal models, therapies to prevent or remove Aβ have generally had limited effects in clinical trials to slow down cognitive decline [3–5]; other approaches are also needed.

Progranulin (PGRN) is a glycosylated protein of 75–80 kDa that can be secreted or transported to lysosomes [6]. It is expressed in many different tissues and cell-types [7]. PGRN protein is composed of seven and a half repeats of a highly conserved cysteine-containing motif that can be cleaved into granulin peptides (A-G), some with proinflammatory properties [8]. In brain, PGRN has been demonstrated to regulate neuroinflammation [9, 10], neurite branching and outgrowth [11, 12], and lysosomal function [13, 14]. The role of PGRN in AD has attracted attention in recent years since the discovery that mutations in GRN, the gene for progranulin, is one cause of frontotemporal dementia (FTD) resulting from frontotemporal lobar degeneration (FTLD) [15, 16]. In FTD, loss of function mutations in the GRN gene resulting in significantly reduced levels of PGRN protein lead to neurodegeneration [15]. It has been hypothesized that reduced PGRN results in neurodegeneration due to enhanced neuroinflammation [10, 17]. The mechanism of reduced PGRN causing enhanced neurodegeneration in FTD and AD has been investigated using gene deletion rodent models but with conflicting results depending on the model [18–22]. Complete loss of PGRN results in enhanced neuroinflammation and disturbance of lysosomal function, but the clinical phenotypes of mice with heterozygous GRN deletion were variable [13, 23, 24]. Increasing PGRN levels in animal models of FTD, AD and Parkinson’s disease (PD) have been reported to reduce both pathological and clinical features [19, 23, 25–27]. However, there are increased levels of PGRN protein in human AD-affected brains and AD mouse models [19]. It has been suggested that the onset of AD might be caused by a drop in PGRN levels prior to the end-stage increase, but this has only been demonstrated in AD mouse models not human subjects [19]. The single nucleotide polymorphism (SNP) rs5848 (T) allele has been associated with an increased risk of AD due to its effect on PGRN protein levels, but these effects were not large [28, 29]. Biomarker studies of PGRN levels in human cerebrospinal fluid (CSF) and plasma in AD subjects have shown changes with disease progression but limited diagnostic utility [28, 30]. While most experimental studies on PGRN in brain have employed animal models of FTD with single mutation or GRN gene knockout, the number of studies relating to PGRN and AD are limited, but one feature observed in studies of AD transgenic mice and human brain samples was that PGRN accumulated around Aβ plaques [19, 31–34]. An additional study that employed granulin domain-specific antibodies showed immunoreactivity of neurons, microglia and structures associated with plaques [35].

Prosaposin (PSAP) is also a lysosomal regulatory protein with significant neuroprotective properties [36–38]. Recent studies have shown biochemical interactions between PGRN and PSAP, with these interactions affecting the trafficking of these proteins to lysosomes [39–41]. There were reduced levels of PSAP in neurons of GRN-deficient mice and in samples from FTD patients with GRN mutations [42]. Transgenic mice with reduced PSAP expression demonstrated similar pathological and behavioral changes as GRN gene-deficient mice [42]. PSAP deficiencies in mice led to significant impairment of PGRN trafficking to lysosomes but increased circulating levels of PGRN [41]. Experimental models of neuronal injury resulted in increased levels of PSAP in neurons and microglia [38, 42, 43]. The interactions of PGRN and PSAP are complex as both PSAP reduction and overexpression resulted in elevated levels of extracellular/secreted PGRN in different cellular models [4]. Overexpression of PSAP increased the concentration of PGRN oligomers, while PSAP knockdown increased concentrations of PGRN monomers [39]. These interactions affecting the levels, localization and aggregation of PGRN might have significant effects on its different biological activities. A recent proteomics study of CSF identified PSAP as a biomarker to discriminate between preclinical AD and control cases [44].

As a result of the previous reports of increased PGRN expression in AD brains in contrast to the deficits occurring in FTD due to GRN mutations, detailed investigations using immunohistochemistry and biochemical techniques were carried out to address the question how increased expression of PGRN, a documented protective molecule, could be associated with pathology in AD. We employed a series of human brain samples from non-demented cases with low plaque and high plaque pathology, along with samples from demented AD cases with high plaque and tangle pathology to study the progression of changes of PGRN and PSAP expression and their interactions. We identified that PGRN and PSAP expression were increased in AD cases, and their interaction could be demonstrated in human brain samples. The interaction with PGRN and PSAP occurred early in plaque development being detectable in plaques present in the low plaque control cases, and PGRN associated with Aβ plaques in all cases were positive to differing extents for PSAP. Overall, these results suggest that the protective and inflammatory modulating properties of PGRN might not be functional in AD, and PSAP bound-aggregated PGRN associated with plaques might lack the biological activities associated with this protein. This can be the basis for further experimental studies, but could be an important issue when considering PGRN supplementation if the protein becomes sequestered into non-active or pathological forms associated with plaques.

## Materials and methods

### Human brain samples

All human brain tissue samples used in this study were obtained from the Banner Sun Health Research Institute Brain and Body Donation Program (Sun City, Arizona, U.S.A.) as part of the Arizona Study of Aging and Neurodegenerative Diseases (AZSAND) [45]. The operations of the Brain and Body Donation Program have received continuous approval of different Institutional Review Boards (IRB). Current operations have been reviewed by Western IRB (Puyallup, WA, U.S.A.). Tissue studies carried out in the U.S.A. were considered non-human subject research under exemption 4 (C.F.R 46.101). Tissue studies carried out in Japan were approved by Shiga University of Medical Science Ethical Committee (Certificate no. 29–114). A summary of demographic details of cases used in this study is presented in Table 1. The details of cases used for immunohistochemistry are described in Table 1a, while those cases used for protein analysis are described in Table 1b. The cases used for protein analysis were all included in the larger group used for immunohistochemistry.

**Table 1:** Demographic details of cases used in study

A. Demographic information of cases used for immunohistochemistry							
	Gender (M:F)	Mean age ± SD	PMI	APOE4	Plaque score±SEM	Tangle score±SEM	BRAAK score
LP (n = 16)	8/8	84.75 ± 6.96	2.93 ± 0.88	0% (0/32)	2.54 ± 2.12	4.92 ± 2.33	I-IV
HP (n = 15)	7/8	85.62 ± 5.98	2.9 ± 0.64	10.7% (3/28)	10.49 ± 2.55	4.5 ± 1.93	II-IV
AD (n = 14)	6/8	80.64 ± 5.58	3.22 ± 0.98	35.7% (10/28)	13.33 ± 3.15	12.46 ± 3.95	V-VI
B. Demographic information of cases used for protein analysis							
	Gender (F/M)	Mean age ± SD	PMI	APOE4	Plaque score	Tangle score	BRAAK score
LP (*n* = 12)	6/6	85.91 ± 8.93	3.09 ± 1.02	4.5% (1/22)	1.33 ± 1.93	5.43 ± 2.44	I-IV
HP (n = 9)	6/3	87.22 ± 8.22	2.72 ± 0.28	12.5% (2/16)	12.05 ± 1.58	5.38 ± 2.02	II-IV
AD (*n* = 11)	4/7	80.27 ± 3.82	3.79 ± 0.47	36.7% (8/22)	14.36 ± 0.67	13.5 ± 1.96	V-VI

### Human brain autopsy and neuropathological diagnosis

At autopsy, brains were sectioned into 1 cm thick coronal slices. Tissue taken from the right hemisphere of each brain donor was frozen on dry ice, while coronal slices from the left hemisphere were fixed for 2 days in 4% paraformaldehyde or 10% formalin, followed by cryoprotection in phosphate buffered glycol/glycerol solution. Frozen brain slices were stored at − 70 to − 80 °C and retrieved for dissection when samples were required for biochemical studies.

All donated brains received full neuropathological diagnosis including reference to pre-mortem clinical history of each case. Consensus clinical and neuropathological criteria were used to diagnose AD, Dementia with Lewy bodies (DLB) or Parkinson’s disease (PD) in donated cases [46, 47]. To assess severity of AD pathology in each case, tissue sections from 5 brain regions (entorhinal cortex, hippocampus, frontal cortex, temporal cortex and parietal cortex) were stained with thioflavin-S, Gallyas and Campbell-Switzer histological stains, and assessed semi-quantitatively for the density of neurofibrillary tangles and amyloid plaques. These methods of assessing pathological load are carried out by the neuropathology department of the Banner Sun Health Research Institute Brain and Body donation program on each donated brain as part of diagnostic procedures. In brief, for each case, each brain region was ranked on a scale of 0–3 based on 0 being no plaques or tangles, 1 being few plaques or tangles, 2 being moderate numbers of plaques and tangles and 3 being numerous plaques and tangles. By combining the measures across these 5 brain regions, assessment of total AD pathology can be ranked on an ordinal scale of 0–15 for plaques and tangles [48]. The cases were classified into low-plaque non-demented (LP) (plaque score < 6), high-plaque non-demented (HP) (plaque score 6–14) and AD with dementia (plaque score > 12). The severity of Lewy body pathology as a score of 0–40 was assessed in 10 different brain regions using immunohistochemistry for phosphorylated alpha-synuclein according to the Unified Staging Scheme for Lewy body disorders [49].

### Apolipoprotein E genotyping

Apolipoprotein E genotypes were determined for most cases using a polymerase chain reaction (PCR)/restriction endonuclease fragment polymorphism method employing DNA extracted from cerebellum to discriminate between APOE2, APOE3 and APOE4 alleles [50]. Results in Table 1 are presented as number of APOE4 alleles out of total numbers of APOE alleles identified in each group.

### Immunohistochemistry

Paraformaldehyde or formalin-fixed tissue sections from temporal cortex (middle temporal gyrus) were used for localization of progranulin (PGRN)-positive cells identified with antibody AF2420 (R&D Systems, Minneapolis, MN, U.S.A.), and for colocalization with Aβ peptide and phosphorylated tau, and markers of microglia (IBA-1, CD45), astrocytes (GFAP), endothelial cells (CD31), lysosomal proteins (LAMP-1, CD68, prosaposin, cathepsin D) and others (sortilin, beta-secretase-1 (BACE1), TMEM106B, neurofilaments, synaptophysin) according to our previously published procedures [51, 52]. Antibodies used in this study are listed in Table 2. For this procedure, 25 μm brain sections were processed using a free-floating method. Sections were rinsed three times in phosphate-buffered saline containing 0.3% Triton-X100 (PBSTx) (0.1 M Phosphate buffer, pH 7.4, 0.137 M NaCl, 0.3% Triton-X100 (Nacalai-Tesque, Kyoto, Japan)), and reacted in PBSTx containing 1% hydrogen peroxide (30 min) to remove endogenous peroxidase activity, rinsed three times in PBSTx and then incubated in optimal dilutions of antibody overnight with shaking at room temperature (RT). Sections were then rinsed three times, incubated in biotinylated anti-species immunoglobulin (Vector Laboratories, Burlingame, CA, U.S.A.) at 1:1000 for 2 h at room temperature, rinsed three times and then incubated with avidin-biotin-peroxidase complex (ABC, 1:1000, Vector Laboratories). Localization of bound antibody was visualized using avidin-biotin horseradish peroxidase (HRP) enzyme complex (ABC-Vector Laboratories) histochemistry and nickel ammonium sulfate-enhanced diaminobenzidine-HCl (100 μg/ml) (Dojindo, Kumamoto, Japan) as substrate to produce a dark purple reaction product. To detect a second antigen, reacted sections were quenched in 1% hydrogen peroxide in PBSTx for 30 min, rinsed and then reacted with the second antibody in the same manner. The second antibody was detected using the same procedure, but with diaminobenzidine-HCl (200 μg/ml) without nickel ammonium sulfate as substrate to produce a brown reaction product. Sections were then mounted on microscope slides, counterstained with neutral red, dehydrated and coverslipped with permanent mounting agent.

**Table 2:** Information of primary antibodies used for the study

Antigen	Antibody	Supplier	Cat#.	Species/Type	Application	Dilution
Progranulin	PGRN	R&D	AF2420	Goat/Polyclonal	IHC	1:4000
		Systems			FIHC	1:1500
					WB	1:2000
					IP	2 μg
Amyloid beta	6E10	Biolegend	803001	Mouse/Monoclonal	IHC	1:2000
(1–16)					FIHC	1:1000
					WB	1:1000
CD45	CD45/HI30	Biolegend	304001	Mouse/Monoclonal	IHC	1:2000
					FIHC	1:1000
PHF-Tau (Ser202/Thr205)	AT8	ThermoFisher	MN1020	Mouse/Monoclonal	IHC	1:2000
PHF-Tau	AT180	Thermo	MN1040	Mouse/Monoclonal	FIHC	1:2000
(Thr231)		Fisher			WB	1:2000
IBA1	IBA1	Fujifilm	019–19,741	Rabbit/Polyclonal	FIHC	1:1000
CD31	CD31 JC/70A	Abcam	Ab9498	Mouse/Monoclonal	FIHC	1:500
GFAP	GFAP	BD bioscience	556330	Mouse/Monoclonal	FIHC	1:1000
LAMP1	LAMP1	Sigma	L1418	Rabbit/Polyclonal	FIHC	1:1000
CD68	CD68	Biolegend	916104	Mouse/Monoclonal	FIHC	1:1000
Prosaposin	PSAP	R&D	AF8520	Rabbit/Polyclonal	FIHC	1:2000
		Systems			WB	1:10000
					IP	2 μg
Sortilin [53]	Sortilin NT3	Abcam	Ab16640	Rabbit/Polyclonal	FIHC	1:1000
					WB	1:2000
TMEM106B [34]	TMEM106B	Bethyl Lab	A303-	Rabbit/Polyclonal	FIHC	1:1000
			439A-1		WB	1:1000
Cathepsin D	cathepsin D	Cell signaling	#2284	Rabbit/Polyclonal	WB	1:1000
BACE1	BACE1	R&D	MAB931	Mouse/Monoclonal	FIHC	1:1000
		Systems			WB	1:2000
Pan NF	SMI312	Biolegend	837904	Mouse/Monoclonal	FIHC	1:1000
Synaptophysin	SVP-38	Sigma	S5768	Mouse/Monoclonal	FIHC	1:500
goat IgG	goat IgG	R&D	AB-108-C	Goat/Polyclonal	WB	1:2000
		Systems			IP	2 μg

Multi-color fluorescent confocal immunohistochemistry was carried out to verify cellular co-localization of PGRN-expressing cells with certain other antigenic markers, as described previously [51, 54]. Tissue sections were incubated with optimal dilutions of antibodies at room temperature overnight with shaking. After three washes (10 min each) in PBSTx, sections were incubated with optimal concentrations of fluorescent-labeled secondary antibodies. Bound primary antibodies were detected with Alexa Fluor 488-donkey anti-goat IgG, Alexa Fluor 568-donkey anti-rabbit or anti-mouse IgG or Alexa Fluor 647-donkey anti-mouse IgG or anti-rabbit IgG (all from ThermoFisher, U.S.A.). Sections were counterstained with Sudan Black (1% solution in 70% ethanol for 3 min) to quench tissue auto-fluorescence, and with DAPI (ThermoFisher, U.S.A.) to reveal nuclei. Sections were coverslipped with fluorescent mounting media (Vector Laboratories) and imaged using an Olympus FV1000 confocal microscope and system software. Some images were acquired using a Leica SP8 confocal microscope and this is indicated on the appropriate figure legend. All images presented are z-stacks of multiple scans (5 scans). These were examined for saturation using software. For imaging of plaques for fluorescent intensity measurements and three-dimensional imaging, z-stacks were acquired to encompass the entire structure (15–20 scans with step-size of approximately 0.46 μm) using the same laser settings.

### Progranulin and Prosaposin antibody validation

To validate the specificity of the PGRN goat antibody (R&D Systems #AF2420), antibody was incubated overnight at 4 °C with recombinant human PGRN protein (R&D Systems #2420-PG, amino acids 18–593) in a mass ratio of 1:200. Similarly, the PSAP rabbit antibody (R&D Systems #AF8520) was incubated with recombinant human PSAP protein (Sino-Biologicals, Beijing, China, #16224-H08H) in the same ratio. Control and protein-absorbed antibodies were diluted to the optimal concentrations for immunohistochemistry and reacted with sections using the above-described enzyme immunohistochemistry procedure. PGRN-absorbed antibody prepared in the same manner was also used for western blots.

### Quantification of Progranulin-positive plaques

To quantify numbers and areas of PGRN-positive plaques, brain sections double-stained for PGRN and Aβ by two-color DAB enzyme histochemistry were used. Photomicrographs were taken with a 10x objective in 3-fields per case. Images were enhanced to maximize color separation between PGRN immunoreactivity (purple) and Aβ plaques (brown). Field selection was performed by choosing 3 evenly-spaced fields encompassing all of the cortical grey matter layers of each case. Images were imported to Adobe Photoshop software (Adobe Inc., San Jose, CA, U.S.A.) and a grid layer consisting of 90,000 pixels per area (field) was created, and 10 fields were measured for a total area of 900,000 pixels/case. The following measures for each section were made; total number of plaques, number of PGRN-associated-plaques, percentage area covered by PGRN-associated plaques and mean area of PGRN-associated plaques as pixels/field.

### Quantification of co-localization of Progranulin and Prosaposin with Aβ plaques

The amounts of colocalization of PGRN- and PSAP-immunoreactivity associated with Aβ plaques were quantified using double-stained confocal sections (PGRN and PSAP) for 3 LP, 3 HP and 3 AD cases. Sections were imaged using an Olympus FV1000 confocal microscope. The settings for laser intensities and number and thickness of scans were determined based on optimal results for LP cases, and these settings were maintained for all sections of HP and AD cases. The images were analyzed using EzColocalization plugin for ImageJ image analysis software [55, 56]. This was used to determine the Pearson colocalization coefficient for 6 separate plaques analyzed for each case (total no. plaques analyzed =18/disease group). Using the same images, the fluorescent intensities of PGRN and PSAP and areas of PGRN/PSAP plaques were also measured for each case. Three-dimensional imaging and colocalization of PGRN and Aβ interactions, and PGRN and PSAP interactions, were carried out using Imaris 8 (Bitplane AG, Switzerland) and Meshlab v2016_12 software (www.meshlab.net).

### Western blotting

Extracts from brain samples were prepared by sonicating each sample in 5-volumes of RIPA buffer (ThermoFisher Scientific; 20 mM Tris-HCl, pH 7.5. 150 mM NaCl, 1% NP40, 1% sodium deoxycholate, 0.1% sodium dodecyl sulfate) supplemented with protease and phosphatase inhibitors (Nacalai-Tesque). These samples were used without centrifugation for preparation of total protein extracts for western blotting. A similar procedure was used to extract proteins from cell pellets of THP-1-derived macrophages and PGRN-overexpressing HEK-293 cells. Total protein concentration of each sample was determined using a MicroBCA assay kit with bovine serum albumin as standard. For SDS gel electrophoresis of brain protein samples, brain protein extracts (1 μg/ul) were dissolved in 4xSDS gel sample buffer (Wako Chemicals-FujiFilm, Japan) with or without reducing agent (0.1 M dithiothreitol), heated to 95 °C for 10 min and loaded onto 4–20% gradient pre-cast gels (Nacalai-Tesque, Kyoto, Japan). Gel electrophoresis was carried out at 100 V in Tris-glycine buffer except for Aβ proteins, which employed Tris-tricine buffer (pH 8.5, 100 mM Tris, 100 mM tricine and 0.1% SDS) as gel running buffer. Separated proteins were transferred to nitrocellulose and processed for detection. Membranes were blocked in 5% skimmed milk dissolved in Tris-buffered saline with 0.1% Tween 20 (TBST – 20 mM Tris-HCl, pH 7.6, 150 mM NaCl, 0.1% Tween 20) and incubated in optimal dilutions of antibody (see Table 2) in 2% milk in TBST overnight at room temperature. Membranes were washed 3 times with TBST and incubated 2 h in HRP-labeled anti-goat, rabbit or mouse IgG (ThermoFisher) at 1:10,000. After a further 3 washes, membranes were exposed to Chemi-Lumi One Super Chemiluminescent substrate (Nacalai-Tesque) and imaged using an ImageQuant LAS 4000 system (GE LifeSciences, U.S.A.). Images were adjusted and band intensities measured using Image Studio Lite software (LI-COR, Lincoln, NE, USA). After initial detection, all membranes (except the immunoprecipitated samples) were reprobed with an HRP-conjugated antibody to β-actin (Abcam, Cambridge, MA. USA) for normalization purposes.

### Paraformaldehyde fixation of Western blot membranes

Increased sensitivity and resolution of PGRN-immunoreactive bands were obtained when western blot membranes were fixed in paraformaldehyde (PFA) vapor. A modification of the procedures described to increase detection of α-synuclein was used [57–59]. Dried membranes were exposed to vaporized PFA, rather than by immersion fixation, in a sealed chamber at 60 °C for 30 min. After the PFA vapors had been vented, the membranes were processed using the above-described western blot detection method.

### Enzymatic Deglycosylation

Enzymatic deglycosylation of PGRN was performed according to the manufacturer’s protocol using PNGase F (New England Biolabs, Beverley, MA, U.S.A.) [54]. Twenty μg of protein lysates from cells and brain samples were diluted in denaturation buffer and heated at 100 °C for 10 min. PNGase F enzyme (1 unit) was added to those samples along with enzyme buffers and NP-40. Following incubation of 1 h at 37 °C, samples were diluted in 4xSDS sample buffer and analyzed by the described SDS-PAGE/western blot method.

### Co-Immunoprecipitation

RIPA-brain extracts prepared for western blots were centrifuged at 14,000 g/30 min to prepare samples for immunoprecipitation assays. Immunoprecipitations were carried out using protein G- or protein A-coupled magnetic beads (G-Biosciences, St. Louis, MO, U.S.A.) conjugated with test antibodies. For each sample, 10 μl of Protein G or Protein A magnetic beads were collected and washed with PBS 0.01% Tween 20 (PBST) using a magnetic stand, which were then mixed with 2 μg of antibody (PGRN goat polyclonal, PSAP rabbit polyclonal, or normal goat IgG) for 30 min with constant mixing. Unbound antibodies were removed by washing beads with PBST, then 200 μg of brain protein extract or 100 μg of cell protein extracts were added to the antibody-coupled beads. Samples were mixed with antibody-conjugated beads for 18 h at 4 °C with rotation, washed three times with RIPA, and then eluted into SDS sample buffer without reducing agent at 80 °C. The omission of reducing agent and lower denaturation temperature prevented the eluted immunoglobulin molecules from being denatured to molecular sizes that interfere with detection of target proteins. Samples were separated through SDS polyacrylamide gels and transferred to nitrocellulose membranes as described and detected with different antibodies by western blot.

### Progranulin-expressing cell culture

Protein extracts from macrophage-like cells produced from the THP-1 monocytic cell line and neuronal cells produced from LAN-5 neuroblastoma cells were used PGRN-containing samples for antibody validation studies,. THP-1 monocytes (TIB-202) obtained from the American Type Culture Collection (Manassas, VA, U.S.A.), were cultivated in suspension culture using RPMI media (Nacalai-Tesque) supplemented with 10% fetal bovine serum (FBS), and differentiated into adherent macrophage-like cells by treatment with 25 nM phorbol myristate acetate (PMA – Sigma Aldrich, St. Louis, MO, U.S.A.) for 3 days in RPMI with 5% FBS. LAN-5 neuroblastoma cells (provided by Dr. R.C. Seeger, Children’s Hospital of Los Angeles, CA, U.S.A.) were used as a human neuronal model [60]. Cells were cultured in RPMI with 10% FBS and differentiated in RPMI with 5% FBS containing 10 μM retinoic acid (Nacalai-Tesque). A PGRN-overexpressing stable-transfected HEK cell line was also prepared. HEK cells were transfected with plasmid expressing PGRN protein fused with a green fluorescent protein sequence (gift from Dr. Morimura, Shiga University of Medical Science, Japan) and selected for resistance to G418 (500 μg/ml). Cells were collected and analyzed by western blot or immunoprecipitation for expression of PGRN.

### Data analysis

Western blot data and plaque measurement data were analyzed by one-way Analysis of Variance (ANOVA) with Newman-Keuls post-hoc test for significance between paired groups. Significant differences were assumed if *P* values of less than 0.05 were obtained. All statistical analyses were carried out using Graphpad Prism Version 7 software (Graphpad software, La Jolla, CA, U.S.A.).

## Results

### Progranulin (PGRN) immunoreactivity in AD pathological structures

**Fig. 1:**
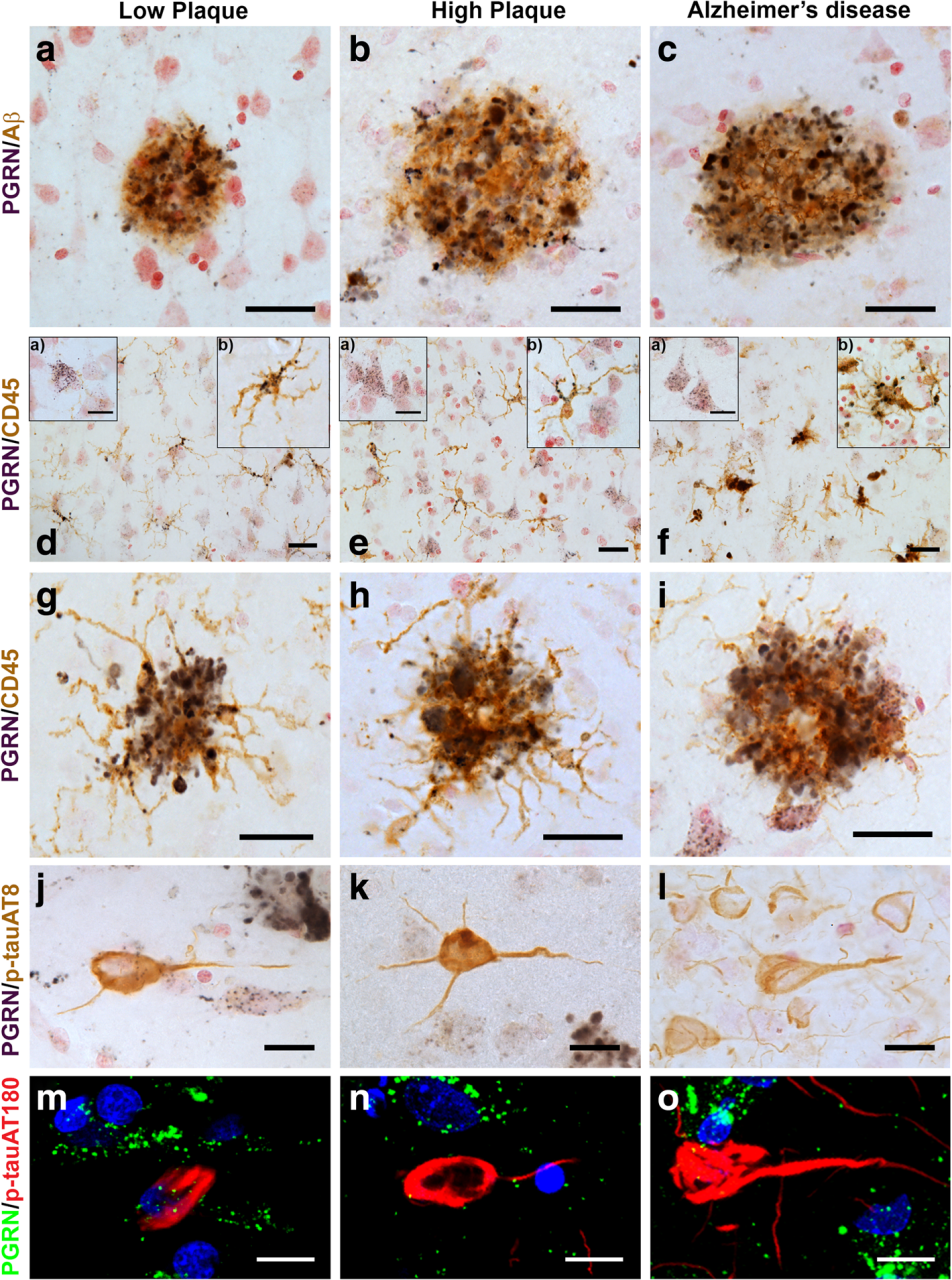
Progranulin Interactions with AD pathological Features. (**a-c**). Representative photomicrographs of progranulin (PGRN)(purple) immunoreactivity associated with amyloid beta (Aβ) plaques (brown) in MTG sections of low plaque, high plaque and Alzheimer’s disease cases. Scale bar represents 30 μm. (**d-f**). Photomicrographs of PGRN (purple) immunoreactivity associated with CD45 immunoreactive microglia in MTG sections of low plaque (**d**), high plaque (**e**), and Alzheimer’s disease cases (**f**). Insets a) show at higher magnification PGRN-positive stained neurons present in each section. Neurons are identified by their size and characteristic shape. Insets b) show higher magnification of PGRN-positive microglia. Scale bar represents 20 μm (**d-f**), and 10 μm for insets. (**g-i**). Photomicrographs of PGRN (purple) with plaque-associated CD45-positive microglia (brown). Progressive increase in accumulation of CD45-positive microglia in low plaque (**g**), high plaque (**h**) and Alzheimer’s disease (**i**) cases. Scale bar represents 30 μm. (**j-o**). Absence of PGRN immunoreactivity of neurofibrillary tangles. (**j-i**) Photomicrographs of PGRN (purple) and phosphorylated tau (AT8)(brown) double-stained sections from low plaque (**j**), high-plaque (**k**), and Alzheimer’s disease cases (**l**). (**m-o**). Confocal micrographs of PGRN (green) and phosphorylated tau (AT180)-positive tangles in low-plaque (**m**), high-plaque (**n**) and Alzheimer’s disease (**o**) cases. Scale bar represents 10 μm.

Initial analysis of PGRN expression in relation to pathological structures in human middle temporal gyrus (MTG) were carried out using dual-color enzyme immunohistochemistry on free-floating (25 μm) sections. The complete series of low plaque non-demented (*n* = 16), high plaque non-demented (*n* = 15) and AD cases (*n* = 14) were stained for PGRN in combination with 6E10, an antibody that detected Aβ, and PGRN in combination with CD45, a marker to identify microglia. Figure 1-panels A-C show representative images of the morphologies of PGRN-associated with Aβ plaques. In low plaque (low pathology) non-demented cases, it was observed that although plaque numbers were sparse, many were PGRN-positive (Fig. 1a). The size and number of PGRN-associated plaques increased in the high plaque and AD cases (Fig. 1b, Fig. 1c).

It was hypothesized that PGRN immunoreactivity around plaques would be due to its expression by infiltrating microglia. PGRN/CD45 stained sections revealed that most microglia were positive for PGRN, but this was more intense in reactive microglia in AD cases (Fig. 1d-f). PGRN immunoreactivity was also detected in neurons in these sections. These could be clearly identified by their morphology (Fig. 1d-f, insets a). The morphology of individual microglia are shown as insets b in Fig. 1d-f. Figure 1 panels G-I show that PGRN-immunoreactive plaque structures are infiltrated by microglia in each of the disease groups, but much of this PGRN immunoreactivity was not associated with microglia. Selected sections from these staged plaque series with low, medium and abundant numbers of tangles (Table 1) were also stained for PGRN and phosphorylated tau (p-tau). Two separate p-tau antibodies were used; AT8, which is specific for tau phosphorylated at serine 202 and threonine 205, and AT180, which is specific for p-tau at threonine 235. Figure 1 (panels J-L) show that AT8 immunoreactive tangles were not positive for PGRN, and similarly, using confocal microscopy AT180 immunoreactive structures were not positive for PGRN (Fig. 1, panels M-O).

### Progranulin is expressed in neurons, microglia and blood vessels but not astrocytes in human middle temporal gyrus

**Fig. 2:**
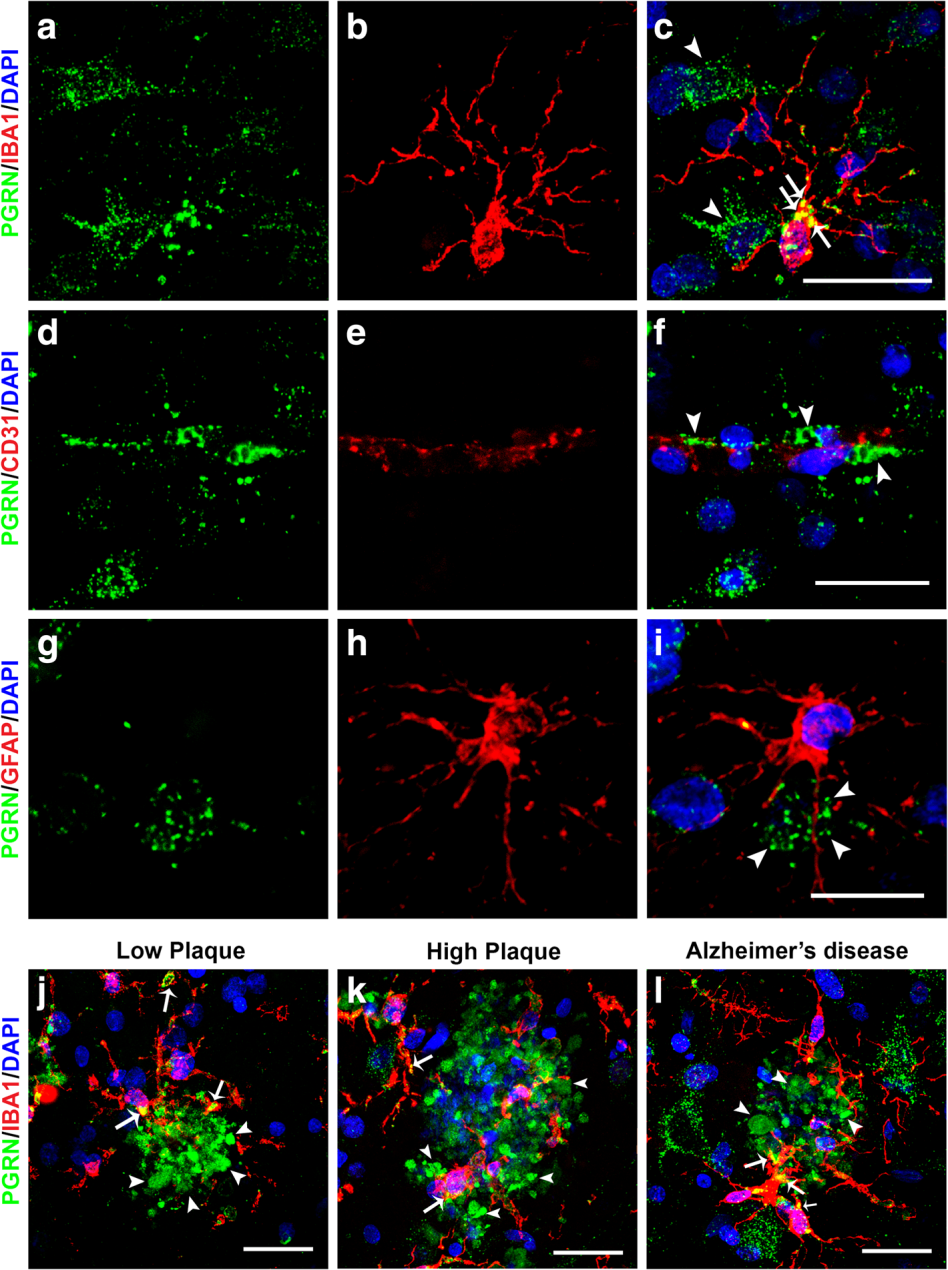
Confocal Immunohistochemistry of progranulin with cellular markers. (**a-c**). Cellular expression of PGRN (green) (**a**), microglial marker IBA-1 (red) (**b**) and colocalization of structures within microglia (yellow) (**c**) in a high plaque case. Green immunoreactivity that did not colocalize with IBA-1 (red) immunoreactivity identified cells with neuronal morphology. Scale bar represents 20 μm. (**d-f**). PGRN immunoreactivity (green) (white arrowheads) was present within cerebral vessels, but did not colocalize with the endothelial marker CD31 (red) in AD case. Scale bar represents 20 μm. (**g-i**). PGRN immunoreactivity (green) (**g**) did not colocalize with astrocyte marker GFAP (red) (**I**). Scale bar represents 20 μm. (**j-l**). Expression of PGRN (green) and microglial marker IBA-1 (red) associated with plaque structures of low plaque (**J**), high plaque (**k**), and Alzheimer’s disease (**l**). PGRN-positive microglia (yellow – arrows) are observed in microglia clustering and infiltrating PGRN- positive plaque-associated structures. Most of the PGRN immunoreactivity (arrowheads) appeared separate from DAPI-stained nuclei (DAPI – blue). Scale bar represents 30 μm.

To confirm colocalization features at the cellular level, multi-color confocal fluorescent microscopy was carried out on selected cases from each group. Colocalization of PGRN in microglia was confirmed by confocal microscopy with microglia being identified using an antibody to IBA-1 (Fig. 2, panels A - C). A feature of PGRN immunoreactivity that can be seen in Fig. 2 (panels A and C) is PGRN immunoreactive structures in microglia (arrows) are larger than those in neurons (arrowheads). We had also observed vascular staining for PGRN in enzyme histochemistry-stained sections, but using confocal microscopy, we could not confirm that immunoreactivity colocalized with CD31, a specific marker for endothelial cells (Fig. 2f, arrowheads). PGRN staining was present in many vessels with staining possibly being in the extracellular basement membrane surrounding the vessels (Fig. 2, panels D-F). Staining of PGRN in brain vasculature has not previously been reported. PGRN immunoreactivity did not colocalize with GFAP-positive astrocytes in any of the sections examined (examples; Fig. 2, panels G-I). We also further examined the interaction of IBA-1-positive microglia with PGRN in low plaque, high plaque and AD cases (Fig. 2, panels J-L). Most plaque-infiltrating microglia showed some colocalization with PGRN (yellow, indicated by arrows), but these figures show that most plaque-associated PGRN did not colocalize with microglia (arrowheads).

### Validation of progranulin antibody used for immunohistochemistry

Due to concerns raised about the specificity of commercial antibodies to PGRN and granulin (GRN) [61], validation studies were carried out with the PGRN antibody (AF2420; goat polyclonal, R&D Systems, Minneapolis, MN, U.S.A.) used in this study. This antibody has unique features having being prepared against a glycosylated recombinant fragment consisting of the majority of the PGRN protein (amino acids 18–593). Immunization with this peptide will have produced an antibody specific against many different epitopes encompassing the complete PGRN protein rather than a single epitope.

Immunohistochemistry was carried out with MTG tissue sections using PGRN antibody that had been absorbed with PGRN-immunizing peptide or control-absorbed. Sections were developed for single-color enzyme immunohistochemistry with counterstaining. Additional file 1: Figure S1 illustrates results from one of the AD sections examined showing that immunohistochemistry with absorbed antibody resulted in absence of cellular and plaque staining (Additional file 1: Figure S1A compared to Figure S1B).

Further characterization of the PGRN antibody was carried out by western blot using protein extracts of MTG brain samples and PGRN-expressing cells. This antibody detected major polypeptide band(s) of approximately 75–80 kDa, which corresponds to full-length glycosylated PGRN, and also a minor polypeptide band of 55 kDa. In samples with high levels of PGRN expression, higher molecular immunoreactive bands can also be detected (Additional file 2: Figure S2A and Figure S2B) suggestive of dimers. Western blots showed that the PGRN polypeptide bands were absent in blots probed with peptide-absorbed (+ Peptide) antibody compared to unabsorbed (−Peptide) antibody (Additional file 2: Figure S2A). Optimization studies for the detection of PGRN polypeptides by western blot showed that omission of reducing agents in brain and control cellular samples significantly enhanced the sensitivity of detection of PGRN polypeptides (Additional file 2: Figure S2B). This was particularly noticeable in extracts of THP-1 macrophage-like cells and LAN-5 neuronal-like cells (Additional file 2: Figure S2B, comparing between + DTT and – DTT lanes). Furthermore, fixation of western blot membranes with paraformaldehyde (PFA) vapor also increased the resolution and sensitivity of the detected PGRN bands in brain samples. The major polypeptide band(s) of 75–80 kDa could be resolved into two separate bands on the PFA-treated membranes (Additional file 2: Figure. S2C). This pretreatment allowed better detection of the 55 kDa band that could be difficult to detect in brain samples of non-PFA treated membranes. Subsequent western blot analyses incorporated these technical changes (Fig. 6 and Additional file 3: Figure S3). To show that the 75–80 kDa bands were glycosylated and the 55 kDa band represented unglycosylated PGRN, brain samples and THP cell extracts were treated with the deglycosylation enzyme PNGaseF and analyzed by western blot (Additional file 2: Figure S2D). The shift from 75 kDa to 55 kDa was evident in only one of two bands in the PNGaseF-treated samples, but increased levels of 55 kDa in these brain samples can be clearly seen. In the treated THP cell extract, the shift in molecular weight was complete (THP+ compared to THP- sample) (Additional file 2: Figure S2D).). These findings verified that the goat PGRN antibody specifically identified PGRN in tissue and in protein extracts. An important feature to observe in Additional file 3: Figure S3A)., which shows the complete membranes used for measurements of PGRN in brain samples, is the absence of low-molecular weight granulin peptides.

### Characterization of progranulin immunoreactive structures associated with plaques in staged MTG cases

**Fig. 3:**
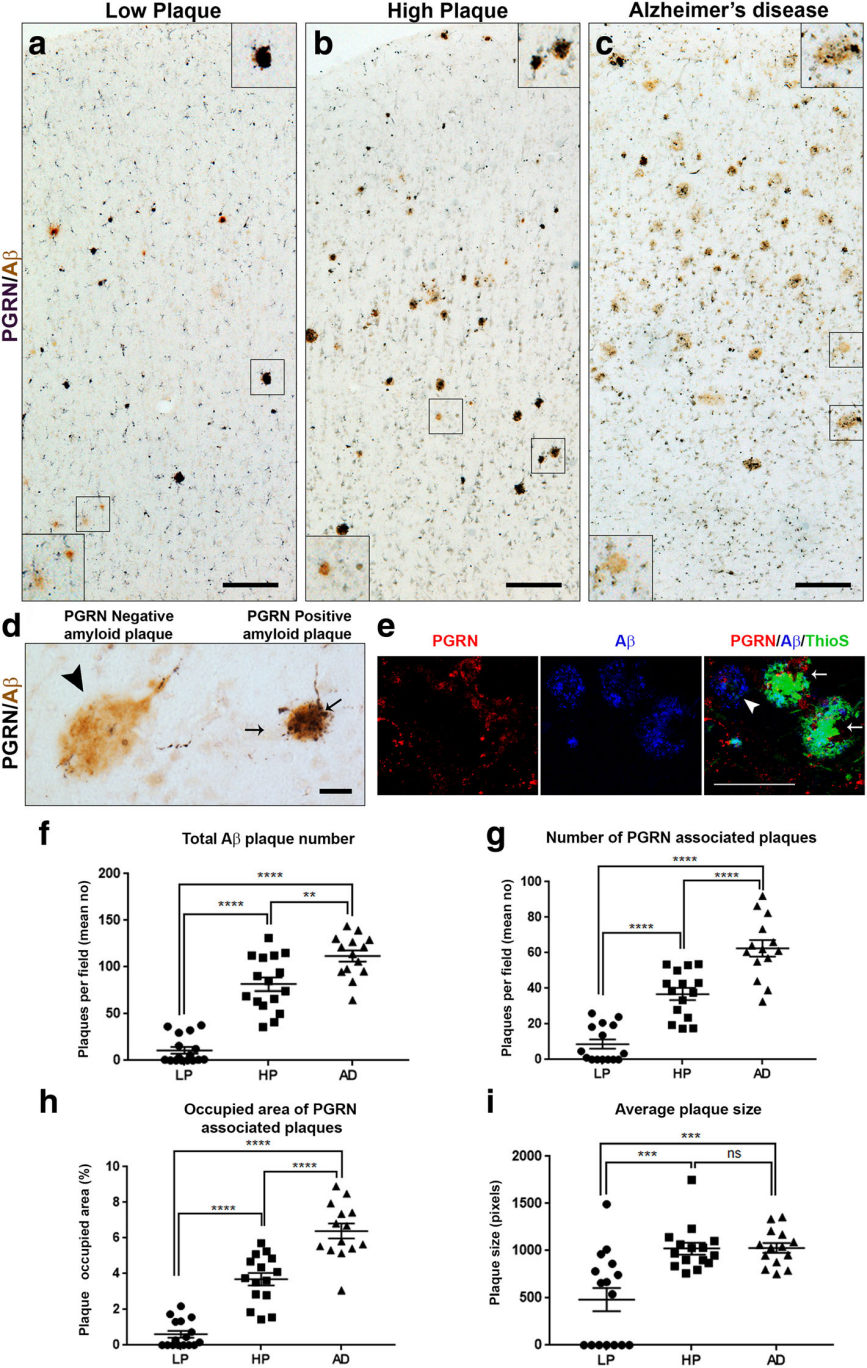
Quantification of numbers and areas of progranulin-positive plaques. (**a-c**). Low magnification of representative images of low plaque (**a**), high plaque (**b**) and Alzheimer’s disease (**c**) cases stained for PGRN (purple) and Aβ (6E10) brown. Series of sections were used for quantification using image analysis software. Examples of PGRN-positive (top) and PGRN-negative plaques (lower) are shown as insets. Scale bars represent 200 μm. (**d**). Higher magnification images of PGRN-negative (arrowhead) and PGRN-positive (arrows) amyloid plaques identified with antibodies to PGRN (purple) and 6E10 (brown). PGRN-positive plaques tended to have a more aggregated morphology while PGRN-negative were more diffuse. Scale bar represents 30 μm. (**e**). PGRN, Aβ and thioflavin-S confocal microscope image. Colocalization of PGRN (red) associated with thioS-positive (green)-Aβ plaques (blue)(arrows) with thioS-negative Aβ plaque with minimal amount of PGRN (arrowhead) in an AD section. Scale bar represents 20 μm. Images were acquired with Leica SP8 microscope. **(F)**. Bar chart showing total number of Aβ plaques in all cases studies. LP (*n* = 16), HP (*n* = 15) and AD (*n* = 14). Results represent mean ± standard error of mean (S.E.M.). Significant increase in numbers of plaques in HP and AD compared to LP **** (*p* < 0.0001), between LP and HP **** (p < 0.0001) and between HP and AD (** *p* < 0.01). **(G)**. Bar chart showing number of Aβ plaques with associated PGRN in all cases studies. LP (n = 16), HP (n = 15) and AD (n = 14). Results represent mean± standard error of mean (S.E.M.). Significant increase in numbers of plaques in HP and AD compared to LP **** (p < 0.0001) and between HP and AD (**** p < 0.0001). (**h**). Bar chart showing total area (percentage) occupied by PGRN-associated Aβ plaques in all cases studies. LP (n = 16), HP (n = 15) and AD (n = 14). Results represent mean ± S.E.M. Significant increase in numbers of plaque occupied area in HP and AD compared to LP **** (p < 0.0001) and between HP and AD (**** p < 0.0001). (**i**). Bar chart showing average plaque size (pixels) of PGRN-associated plaques in all cases studies. Numbers derived from total area occupied divided by number of plaques for each case. Results represent mean ±S.E.M. LP (n = 16), HP (n = 15) and AD (n = 14). Significant increase in average plaque size in LP and AD compared to LP ****p < 0.001) and between HP and LP (*** p < 0.001). NS - Not significantly different.

It was observed that many of the Aβ plaques present in all cases studied had PGRN-immunoreactive structures, even the few plaques present in many of the low plaque cases. This indicated that PGRN association with Aβ plaques was an early pathological event. The distribution of PGRN and Aβ immunoreactivity across cortical layers is shown at low magnification in representative images of low plaque (Fig. 3a), high plaque (Fig. 3b), and Alzheimer’s disease (Fig. 3c) cases. Inset images represent PGRN-positive and negative plaques in each group. Examples of PGRN-negative and positive plaques are also shown at higher magnification (Fig. 3d). The negative plaques appeared to have a more diffuse morphology than positive plaques. This was characterized by staining selected sections with thioflavin-S (thio-S) to identify aggregated plaques. PGRN immunoreactivity was associated with thio-S positive, Aβ positive plaques but not thio-S negative, Aβ positive plaques (Fig. 3e). Seven of the 15 LP cases had no detectable Aβ plaques in the MTG sections examined, while in the remaining LP cases with Aβ plaques, many of these were PGRN-positive. The numbers of Aβ plaques (Fig. 3f) and the number of PGRN-associated Aβ plaques (Fig. 3g) were calculated in sections from each case. This measure did not account for the size or intensity of PGRN immunoreactivity in plaques only its presence or absence. We used these images to calculate the percentage area occupied by PGRN-associated Aβ plaques for each case (Fig. 3h), and the mean area of positive plaques (Fig. 3i). Although there were significantly more PGRN-positive plaques with significantly larger total areas in the AD cases compared to the HP cases, we noticed differences in the morphologies of PGRN-associated Aβ plaques between HP and AD cases. Many of the PGRN-positive Aβ plaques in AD cases had less intense coverage of PGRN immunoreactivity (top insets Fig. 3b and c). This was investigated further in subsequent sections.

**Fig. 4:**
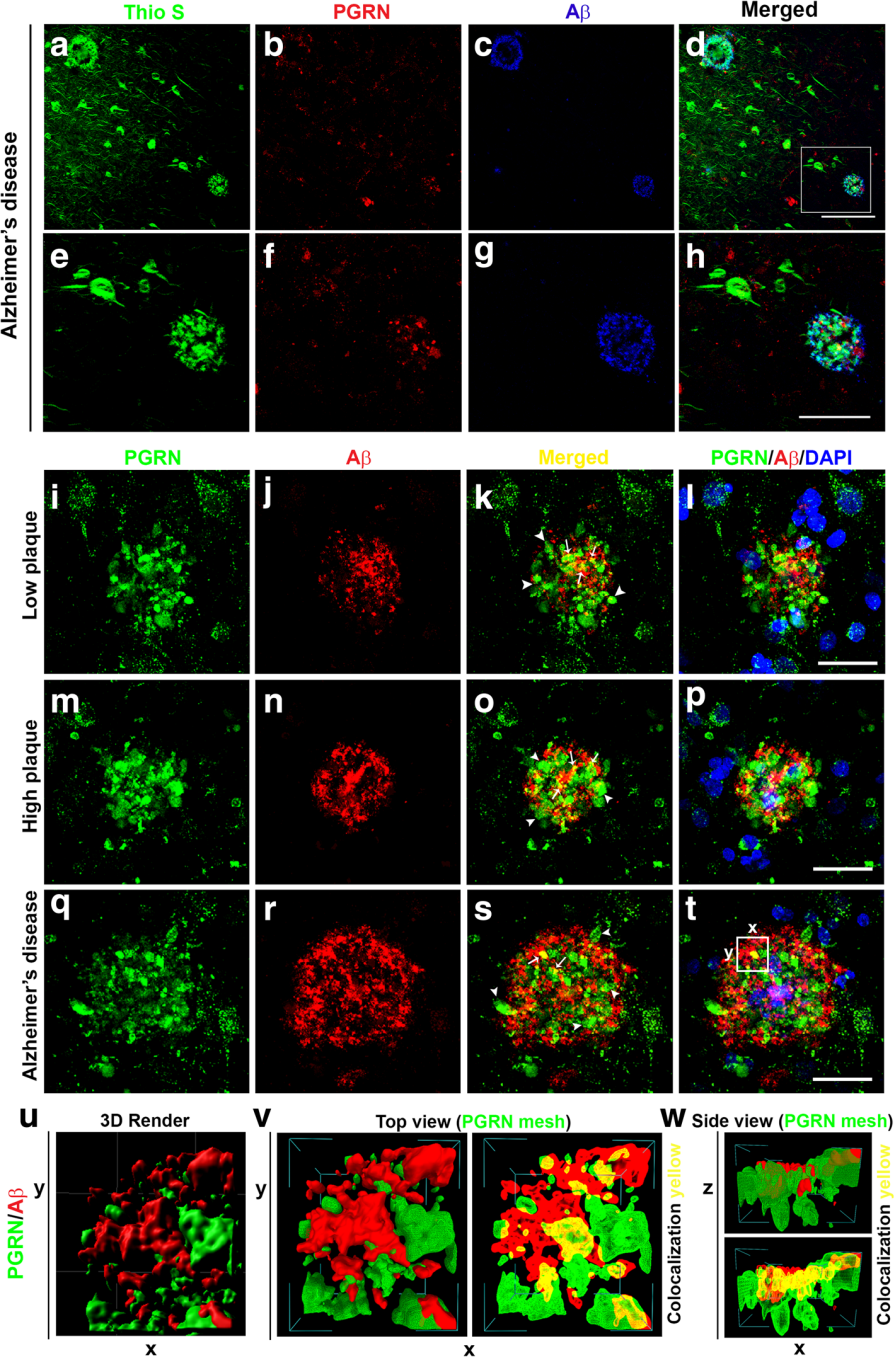
Characterization of progranulin immunoreactivity associated with different types of Aβ plaques in low plaque, high plaque and Alzheimer’s disease cases (**a-d**). Thioflavin-S staining of Alzheimer’s disease tissue section (low magnification) characterization of PGRN (red) and Aβ- immunoreactive plaques (blue). Triple-color merged image (D) identifies that thio-S plaques have PGRN-positive associated structures but thio-S positive tangles show no PGRN immunoreactivity. Box area in D illustrates areas shown at higher magnification in panels E-H. Scale bar represents 100 μm. (**e-h**). Higher magnification of area from D showing thio-S positive plaques and tangles (green), PGRN (red) and Aβ (blue). Scale represents 25 μm. Images were acquired with Leica SP8 microscope. (**i-l**). Low Plaque case: Features of PGRN (green) immunoreactive accumulations (**I**) around Aβ (red) immunoreactive plaque (**j**), and merged images (**k** and **l**) showing limited overlap (yellow) between PGRN and Aβ. Image D with DAPI-stained nuclei demonstrate the presence of cells around the plaque. (**m-p**). High Plaque case: PGRN (green)(M) and Aβ (red)(N) immunoreactivity showing more extensive colocalization (yellow) (O and P). (**q-t**). Alzheimer’s disease case: PGRN (green) (**q**) immunoreactivity and Aβ (red) (R) show less colocalization (yellow) with the formation of larger AD plaques. PGRN aggregates are present within the Aβ immunoreactive areas but with limited colocalization (Q and T). Three-Dimensional Reconstructions of progranulin-positive Aβ plaques. (**u-w**). Three-dimensional reconstruction and Mesh rendering of PGRN and Aβ immunoreactive plaques in AD case (**U**). Mesh rendering of three-dimensional modelled plaque shown in panel (Top view, panel V). The two immunoreactive structures (PGRN-green; Aβ-red) are present as aggregates but show limited areas of interaction (Side view, panel W). Similar patterns of colocalization were observed for LP and HP cases.

Further detailed studies of PGRN interactions with Aβ plaques were also carried out by confocal microscopy (Fig. 4). Thioflavin-S histochemistry combined with PGRN and Aβ immunohistochemistry was used to show that mature aggregated thio-S positive plaques in Alzheimer’s disease cases showed some associated PGRN immunoreactive structures, while thio-S positive tangles did not (Fig. 4 (a-d), low magnification and Fig. 4 (e-h) higher magnification). Figure 4d (arrowhead) did identify a dense-cored, burnt-out plaque that was thio-S positive but had minimally detectable associated PGRN. The features of PGRN-positive plaques in low plaque, high plaque and Alzheimer’s disease cases revealed that most of the deposits of PGRN around plaques appeared extracellular, and even though cell membranes are not visible in the sections, most PGRN immunoreactivity was not clearly associated with nucleated cells (Fig. 4 panels L, P, T). Comparison of plaque morphologies between cases showed the relative size and distribution of PGRN in plaques varied between groups. In the low plaque and high plaque cases, the PGRN appeared as aggregates around the developing plaque with some colocalization with Aβ (Fig. 4 Merged – yellow arrows Fig. 4k and l compared to Fig. 4o and p). In the AD cases, there was less colocalization, and the Aβ immunoreactivity (Fig. 4s and i) extended beyond the PGRN-positive structures. This feature can also be seen in Fig. 4h).

The confocal images of PGRN and Aβ-stained plaques were further analyzed using computer-assisted three-dimensional imaging. These images showed the interaction of PGRN and Aβ positive structures in the AD case shown in Fig. 4t. Figure 4u shows a computer rendered three-dimensional image of the highlighted area indicated in Fig. 4t. Analysis of the 3-D rendered image with MeshLab software in Top view (Fig. 4v) and Side view (Fig. 4w), which analyzed the interactions through multiple layers, showed some overlap of these structures (Fig. 4v and Fig. 4w – yellow overlap) but most of the structures appeared separate (green: PGRN, red: Aβ). Overall, the arrangements appeared to confirm the images from enzyme and confocal microscopy of the presence of two aggregated structures associated with each other but with limited colocalization. Similar interaction mesh images could be produced from confocal images of PGRN associated with plaques from low plaque and high plaque cases.

Analysis of interactions of PGRN-immunoreactive structures with neuritic plaques were carried out using markers for pTau (antibody AT180), pan-neurofilaments (preferentially phosphorylated neurofilaments) (antibody SMI312) and synaptophysin. Images from Alzheimer’s disease case are shown (Additional file 3: Figure S3 Additional file 3: Figure S3). Each of these markers identify distinct neuronal components. Each neuritic plaque shown (Additional file 3: Figure S3A, D, G) showed strong associated PGRN staining. The merged images showed little to no colocalization of these neuronal markers and PGRN on the neuritic plaques observed (Additional file 3: Figure S3C-pTau; Additional file 3: Figure S3F-NF; Additional file 3: Figure S3I-synapto). This was assessed by the absence of yellow colocalization in the Z-stack images.

### Association of Progranulin with lysosomal proteins

**Fig. 5:**
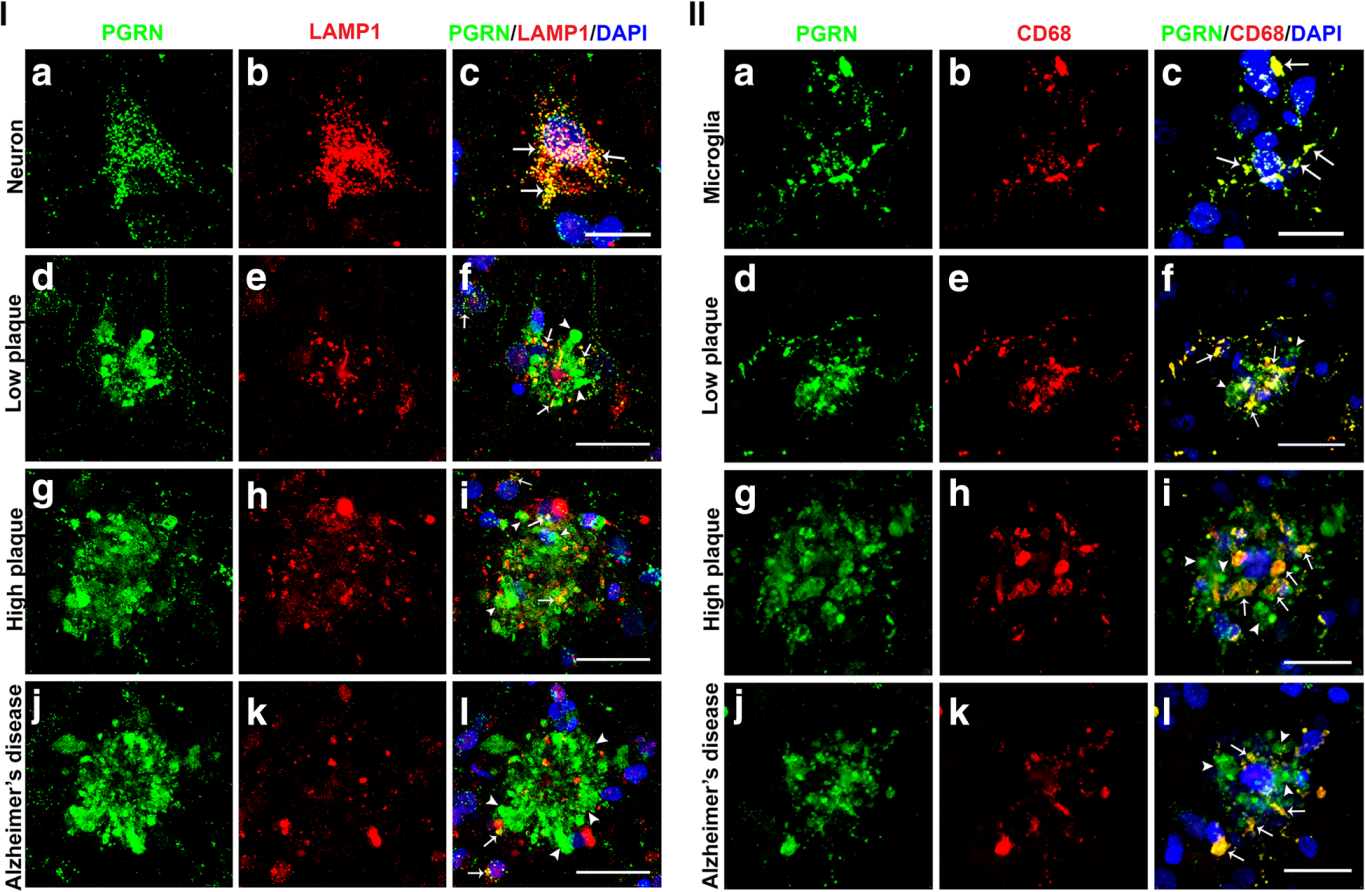
Association of progranulin with lysosomal proteins. **i**) Progranulin and Lysosomal-Associated Membrane Protein-1 (LAMP-1) interactions in neurons and plaques. (**a-c**). PGRN (green) (A) and LAMP-1 (red) (**b**) immunoreactivity in neurons show almost complete colocalization (yellow), (**c**) showing PGRN in neurons is mainly localized to lysosomes. Scale bar represents 10 μm. (**d-f**). PGRN immunoreactivity (green) (**d**) and LAMP-1 (red) (E) in plaques of low plaque case showed limited colocalization (arrows-yellow) (F). Scale bar represents 30 μm. (**g-i**). PGRN immunoreactivity in plaques (green) (G) and LAMP-1 (red) (H) in plaques of high plaque case showed more extensive colocalization (arrows-yellow) (I). Scale bar represents 30 μm. (**j-l**). Limited immunoreactivity for LAMP-1 in AD cases (**k**). PGRN immunoreactivity in plaques (green) (**j**) and LAMP-1 (red) (**k**) in plaques of AD case showed very limited colocalization (arrows-yellow) (**l**). Scale bar represents 30 μm. II) Progranulin and lysosomal protein CD68 interactions in microglia and plaques. **a-c**). PGRN (green) (**a**) and CD68 (red) (**b**) immunoreactivities in microglia show almost complete colocalization (yellow) (**c**) showing PGRN in microglia is mainly localized to lysosomes. Plaque-associated CD68 and PGRN show almost complete colocalization Scale bar represents 10 μm. (**d-f**). Low plaque case: (**g-i**). High plaque case: (**j-l**). Alzheimer’s disease case. CD68 (red) and PGRN immunoreactivity (green) showed extensive colocalization (arrows-yellow) in all groups but most plaque-associated PGRN did not colocalize (green). Scale bar represents 30 μm.

A study of PGRN and lysosomal proteins on plaques of 5xFAD AD-model transgenic mice had observed that most Aβ plaques colocalized with lysosomal proteins, and that most PGRN associated with Aβ plaques colocalized with lysosomal-associated membrane protein-1 (LAMP-1) [32]. These authors hypothesized that PGRN associated with plaques represented an aberrant accumulation in lysosomes. We carried out confocal microscopy with antibodies to lysosomal proteins LAMP-1 and CD68 in conjunction with PGRN to verify whether these observations could be made in human brain materials. Figure 5(I) shows representative results for PGRN and LAMP-1. The antibody to LAMP-1 used in this study only identified neurons and not microglia. Figure 5 (I) (panels A-C) demonstrated that PGRN immunoreactivity almost completely colocalized with LAMP-1 in neurons. This verified the specificity of LAMP-1 immunoreactivity to lysosomal structures. Examining PGRN-positive plaques in sections from low plaque (Fig. 5 (I) (panels D-F), high plaque (Fig. 5 (I) (panels G-I), and Alzheimer’s disease sections (Fig. 5 (I) (panels J-L) identified different amounts of LAMP-1 staining, but limited colocalization of PGRN with LAMP-1 in these plaque structures was evident. There were greater amounts of colocalization in the low plaque and high plaque cases than in the AD cases (Fig. 5 (I) (panels F, I, L) (yellow – indicated by arrows).

CD68, a macrophage-specific lysosomal membrane protein, was used to identify colocalization of PGRN in lysosomes of microglia. Figure 5 (II) (panels A-C) show almost complete colocalization of PGRN and CD68 in isolated microglia on an AD section (yellow – indicated by arrows). Examining plaque structures showed that in low plaque cases, CD68 showed significant colocalization with PGRN (Fig. 5 (II) (panel F). In high plaque and AD cases, with larger plaques, there was still extensive colocalization (yellow-indicated by arrows), but much of the PGRN immunoreactivity did not colocalize with CD68, and appeared not to be cell-associated (green-indicated by arrowheads).

### Progranulin protein levels in low plaque, high plaque and AD MTG samples

**Fig. 6:**
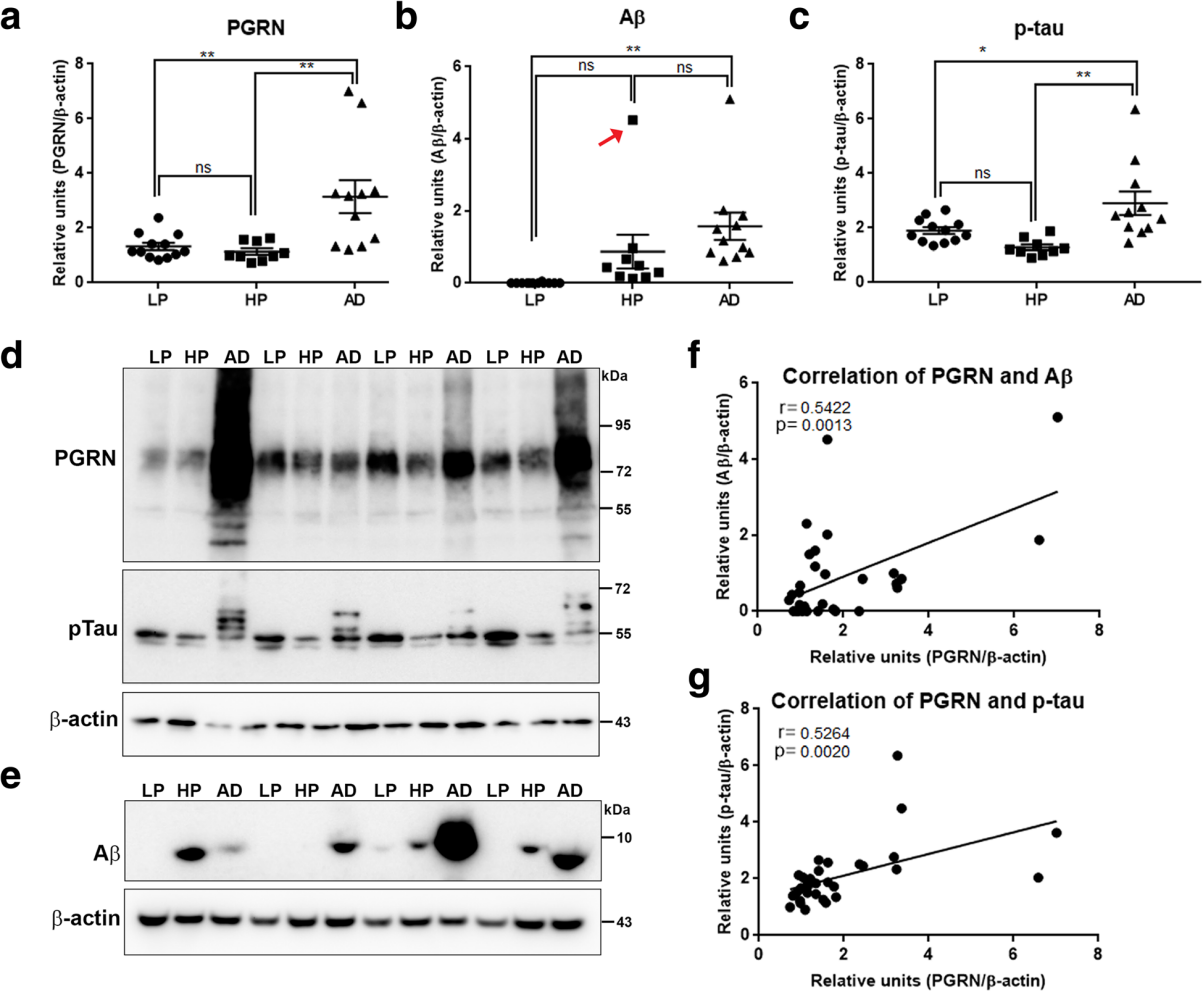
Biochemical analyses of Progranulin in MTG samples compared to Aβ and phosphorylated tau. **a**). Scatter plot showing expression levels of PGRN protein in the different groups. Results presented represent the combination of levels of 75–80 kDa and lower molecular weight (55 kDa) PGRN protein bands. Results represent mean  ±S.E.M. Significantly increased levels of PGRN protein were present in AD case compared to LP and HP cases but not between LP and HP cases (** *p* < 0.01, ns – not significant). **b**). Scatter plot showing expression levels of Aβ protein in the different groups. Results presented represent the levels of all p-tau detected bands. Results represent mean  ±S.E.M. Significantly increased levels of Aβ (monomer) protein were present in AD case compared to LP and AD cases but not between LP and HP or HP and AD cases (** p < 0.01, ns – not significant). Outlier HP case indicated by red arrow (see text). **c**). Scatter plot showing expression levels of phosphorylated tau (p-tau – Thr 231) protein in the different disease groups. Results presented represent the levels of all p-tau detected bands. Results represent mean ±S.E.M. Significantly increased levels of p-tau protein were present in AD cases compared to LP and HP cases but not between LP and HP cases (* *p* < 0.05, ** p < 0.01, ns – not significant). **d**). Western blots showing bands detected with goat antibody to PGRN (PGRN) in MTG protein extracts from low plaque (LP), high plaque (HP) and AD cases. The complete series of samples are presented in Additional file 4: **Fig. S4A**). The same blots were reprobed with antibody AT180 to phosphorylated tau (pTau), and β-actin for normalization purposes. **e**). Western blots showing bands detected with antibody 6E10 to Aβ in MTG protein extracts from low plaque (LP), high plaque (HP) and AD cases. Results represent mean ±S.E.M. Samples were separate from those in panel A by using Tris-tricine gels to resolve low molecular weight bands. **f**). Correlation Analyses between PGRN protein and Aβ protein levels for all samples. Significant correlation (r = 0.5422, *p* = 0.0013). **g**). Correlation Analyses between PGRN protein and p-tau (Thr231) protein levels for all samples. Significant correlation (r = 0.5264, *p* = 0.002).

Measurement of PGRN protein levels in LP, HP and AD samples were carried out using the described technical modifications (omission of reducing agents and paraformaldehyde fixation of membranes) to improve sensitivity of detection of PGRN-related polypeptides. Measurements of total PGRN levels were determined that combined the intensities of the major 75–80 kDa bands and minor 55 kDa band (representative blot Fig. 6d; complete series of blots Additional file 4: Figure S4A). There were significantly increased levels of PGRN in AD cases compared to HP and LP cases, but not between HP and LP cases (Fig. 6a). This indicated the overall increase in PGRN occurred later in the disease. These blots were also analyzed for levels of Aβ (Fig. 6b)–Aβ, and phosphorylated tau (p-tau-Thr231)(Fig. 6c)-pTau). Results showed significantly greater Aβ in AD cases but not compared to HP cases. However, the Aβ level in one HP case (indicated with a red arrow (Fig. 6b) was much higher than the remainder of the HP samples. This sample was from a 99 year-old subject with a plaque score of 14 but without documented dementia. This case had high PGRN levels and was identified as a statistical outlier. Removing it from the analysis of Aβ levels between groups produced significant difference between HP and AD (*p* < 0.01); this case was included in the correlation analyses (Fig. 6f). Correlation analyses between PGRN and Aβ levels (Fig. 6f) showed statistical significance (Pearson r = 0.5422, *p* = 0.0013). We also compared PGRN expression levels with p-tau (Thr231) (Fig. 6d-pTau). Although there was no evidence of p-tau and PGRN colocalization at the cellular level, there was significant correlation between PGRN and p-tau (AT180) levels (Pearson r = 0.5264, *p* = 0.0020) (Fig. 6g). These results indicate total PGRN levels increased with increasing amounts of plaque and tangle pathology.

### Co-immunoprecipitation of Progranulin-binding proteins in brain samples identified prosaposin

**Fig. 7:**
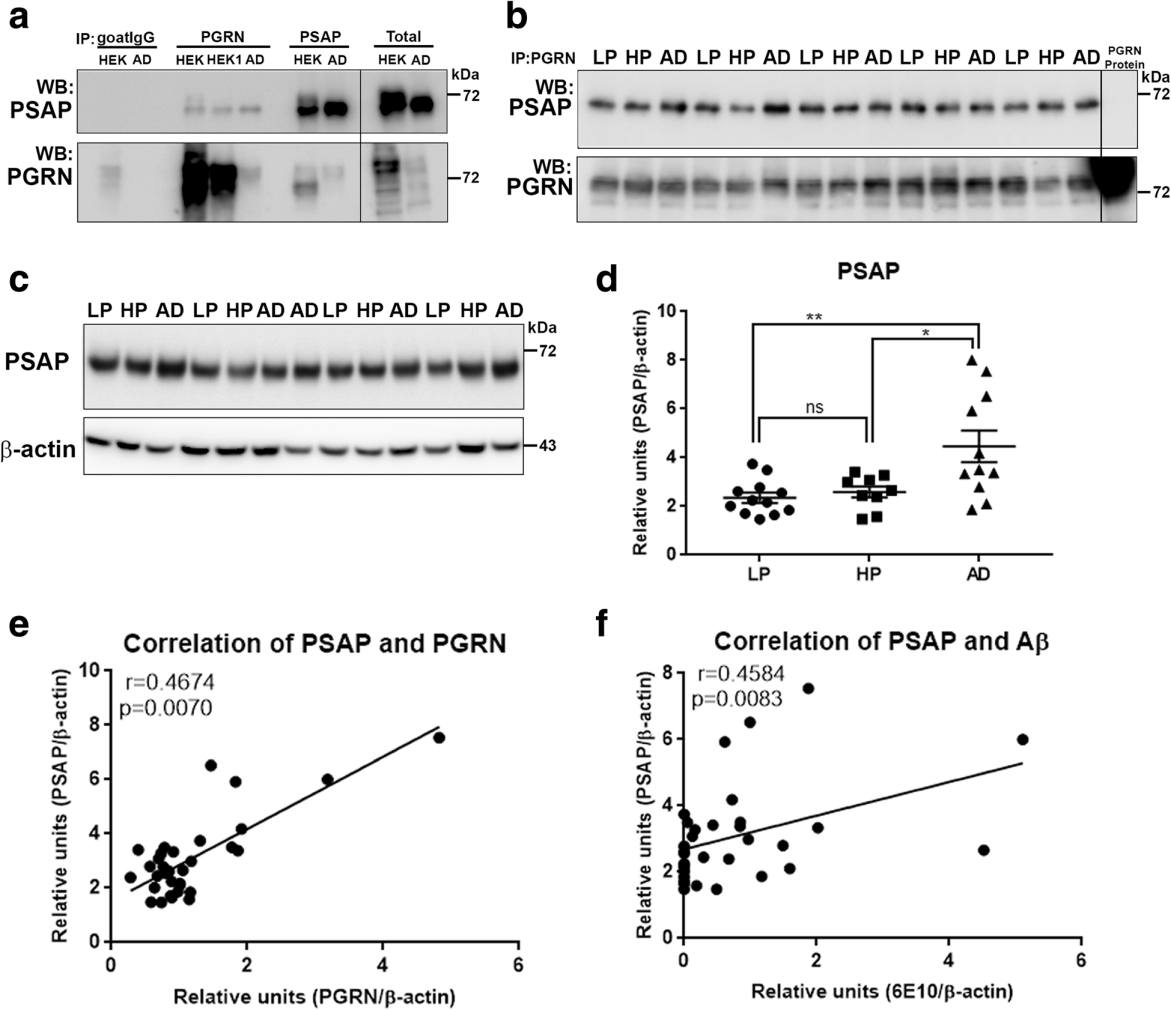
Biochemical analysis of interactions of progranulin and prosaposin in MTG brain samples. (**a**-**b**). Co-immunoprecipitation of Progranulin and Prosaposin. **a**). Western blot control analyses to show interactions of progranulin (PGRN) and prosaposin (PSAP). Immunoprecipitation of PGRN-overexpressing HEK (HEK and HEK1) cells and AD brain sample with protein G- (goat antibody) or protein A- (rabbit antibody) antibody-conjugated magnetic beads. Beads were prepared using non-immune goat IgG, goat anti-PGRN and rabbit anti-PSAP. Immunoprecipitated samples were separated by gel electrophoresis, transferred to membranes, and probed with antibodies to PSAP and PGRN. Samples of total protein (non-immunoprecipitated) from PGRN-overexpressing HEK (HEK) cells and brain sample (AD) were analyzed as specificity controls. Samples immunoprecipitated with PGRN antibody contained PSAP, and samples precipitated with PSAP antibody contained PGRN. **b**). All of the brain samples from LP, AD and HP cases precipitated with PGRN antibody pulled-down PSAP. A series of cases (*n* = 5) from each group were analyzed. Western blot images for both antibodies are shown. (**c-f**) Biochemical analysis of prosaposin in MTG brain protein extracts. **c**). Western blot analysis of total PSAP protein levels in MTG samples. Representative image of western blot demonstrating PSAP protein in samples from LP, HP and AD cases. The complete images of all samples analyzed for PSAP are shown as Additional file 4: Figure. S4B. D). Scatter plot showing expression levels of PSAP protein in the different groups. Results represent mean ±S.E.M. Significantly increased levels of PSAP protein were present in AD case compared to LP and HP cases but not between LP and HP cases. Significant increased levels of PSAP protein in MTG samples from AD compared to HP (* *p* < 0.05) and LP (** p < 0.01). **e**). Positive correlation between PSAP and PGRN protein levels in MTG samples (Pearson r = 0.4674, *p* = 0.0070). **f**). Positive correlation between PSAP and Aβ protein levels in MTG samples (Pearson r = 0.4584, *p* = 0.0083).

We used co-immunoprecipitation/western blot methodology involving precipitation of PGRN from human brain samples with PGRN-antibody conjugated magnetic beads followed by western blot with antibodies to certain previously identified PGRN-interacting proteins. Control experiments identified that immunoprecipitation of PGRN using brain samples and PGRN-overexpressing HEK cells pulled-down prosaposin (PSAP) (Fig. 7a -PGRN). This interaction was confirmed by showing immunoprecipitation of PSAP pulled-down PGRN (Fig. 7a -PSAP). The samples were eluted from the beads under non-reducing conditions and analyzed by western blot for other possible proteins interacting with PGRN. Elution of proteins under non-reducing conditions at 80 °C (instead of 95 °C) resulted in samples with limited interference with dissociated immunoglobulin polypeptides. Under the immunoprecipitation conditions used, we only detected PSAP by western blot in brain samples precipitated with PGRN antibody-conjugated magnetic beads. These samples were also probed with antibodies to sortilin, TMEM106B, cathepsin D and beta-secretase-1 (BACE-1) but did not detect these proteins (Additional file 5: Figure S5A and S5B). However, sortilin, cathepsin D and BACE-1 were detected in brain samples precipitated with PSAP (Additional file 5: Figure S5A and S5B). The lack of colocalization of these proteins with PGRN on plaques was confirmed by confocal microscopy on AD sections (Additional file 5: Figure S5, panels D-O). A larger series of LP, HP and AD brain MTG extracts were analyzed for the interaction of PGRN and PSAP. Samples were immunoprecipitated with PGRN antibody and analyzed for PSAP by western blot (Fig. 7b). Matching blots were probed with PGRN antibody to confirm its precipitation. Quantification of band intensities and calculation of ratio of intensities of PSAP to PGRN showed no significant differences between the different groups of samples (Additional file 5: Figure S5C). Images of the full blots for immunoprecipitation experiments confirm the absence of saposin peptides (Additional file 6: Figure S6A) and granulin peptides (Additional file 6: Figure S6B) interacting with brain samples precipitated with antibody to PGRN. Samples from HEK cells overexpressing PGRN (Additional file 6: Figure S6C- lanes IP PGRN +, HEK) precipitated with PGRN antibody did show some granulin reactive bands in the blot after longer exposure.

To follow up the identification of PSAP as a PGRN-binding protein in brain, quantification of PSAP protein levels was carried out in the same complete series of MTG brain samples used for PGRN western blot analysis. These samples were also analyzed in the absence of reducing agents with proteins on transferred membranes being fixed with PFA vapors, as described for PGRN analyses. These analyses showed a significant increase in AD compared to LP (*p* < 0.01) and HP (*p* < 0.05) cases (Fig. 7d), but not between LP and HP cases. Figure 7c is a representative western blot image of PSAP in brain samples with the complete series of blots shown in Additional file 3: Figure S3B. As expected, there was significant correlation between PGRN and PSAP levels in these brain samples (Fig. 7e - Pearson r = 0.4674, *p* = 0.0070). In addition there was significant correlation between PSAP and Aβ levels (Fig. 7f - Pearson r = 0.4583, *p* = 0.0083).

### Immunohistochemical colocalization of prosaposin with progranulin and cellular markers

We have shown using co-immunoprecipitation methods that PGRN and PSAP are interacting in brain protein extracts. This led to the question whether there were interactions of these molecules around plaques in our human brain samples. Experimental studies have shown that the interactions of PGRN and PSAP have significant effects on their cellular trafficking and biochemical properties [40, 42].

**Fig. 8:**
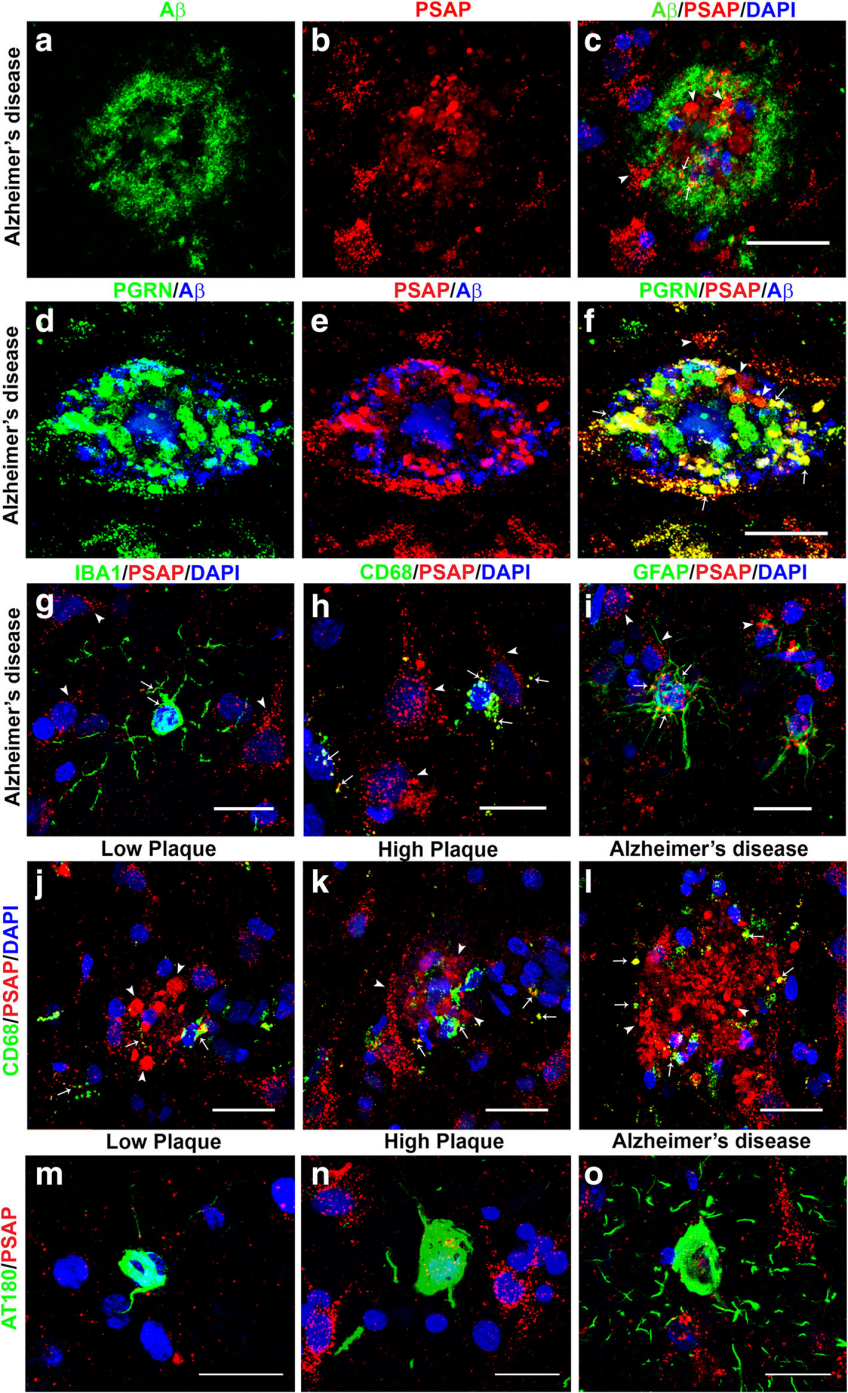
Confocal microscopy of prosaposin localization on plaques and different cell types. (**a-c**). Aβ (green) (**a**) and PSAP (red) plaque (**b**) with limited colocalization (C – yellow) in an AD case. Scale bar represents 30 μm. (**d-f**). Comparison of colocalization in plaque of PGRN (green) and Aβ (blue) (**d**) with PSAP (red) and Aβ (blue) in triple-stained AD section. Merged images show extensive colocalization of PGRN and PSAP (yellow) but limited overlap with Aβ-positive structures. Scale bar represents 30 μm. (**G-I**). Merged images of PSAP (red) immunoreactivity with microglial markers IBA-1 (**g**) and CD68 (**h**) (green) and astrocyte marker GFAP (green) show some expression of PSAP in both cell types (yellow). These images show that PSAP (red) is predominantly in cells with morphology of neurons. Scale bar represents 10 μm. (**j-l**). Merged images of CD68 (green) and PSAP (red) on plaques in low plaque case (**J**), high plaque case (**k**) and AD case (**l**). Significant amounts of PSAP immunoreactivity (red) can be observed on all plaques but with only limited colocalization with CD68 in infiltrating microglia. Scale bar represents 30 μm. **(m-n**) Merged images of AT180 (pTau) (green) and PSAP (red) on tangle in low plaque case (M), high plaque case (**n**) and Alzheimer’s disease case (**o**). Very limited amounts of PSAP immunoreactivity (yellow) can be observed on tangles. Panel M and N show intracellular tangles with DAPI-positive nuclei, while panel O shows extracellular tangle. Scale bar represents 30 μm.

We confirmed the specificity of the PSAP by peptide absorption. Sections reacted with PSAP-absorbed antibody did not show immunoreactivity (Additional file 1: Figure. S1C) compared to control-absorbed antibody (Additional file 1: Figure. S1D). In AD cases, PSAP immunoreactivity was present in most neurons. This is evident in most panels of Fig. 8. PSAP was also associated with many Aβ plaques, but the two molecules seemed to show limited interactions as evidenced by the red immunoreactivity (PSAP) on the plaque and limited amounts of colocalized yellow (PSAP + Aβ) (arrows Fig. 8c)(Fig. 8a-c). Triple staining for PGRN, PSAP and Aβ of plaques in AD cases showed similar morphologies around Aβ-positive structures (Fig. 8d and e). There was extensive though not exclusive colocalization of PGRN and PSAP associated with Aβ-immunoreactive structures (Fig. 8f). As some PGRN staining around plaques can be in microglia and neurons, we determined whether PSAP expression could also be detected in microglia or astrocytes. Double staining of sections for PSAP with IBA-1 and CD68 (microglia markers), and GFAP (astrocyte marker) identified limited amounts of colocalization (arrows Fig. 8g-i), while all images showed that most PSAP immunoreactivity was present in neurons. Double staining for CD68 and PSAP of sections from each group identified plaque-like structures with significant PSAP immunoreactivity (Fig. 8 J-L), but there was only small amounts of colocalization with the CD68-positive microglia infiltrating these plaques.

**Fig. 9:**
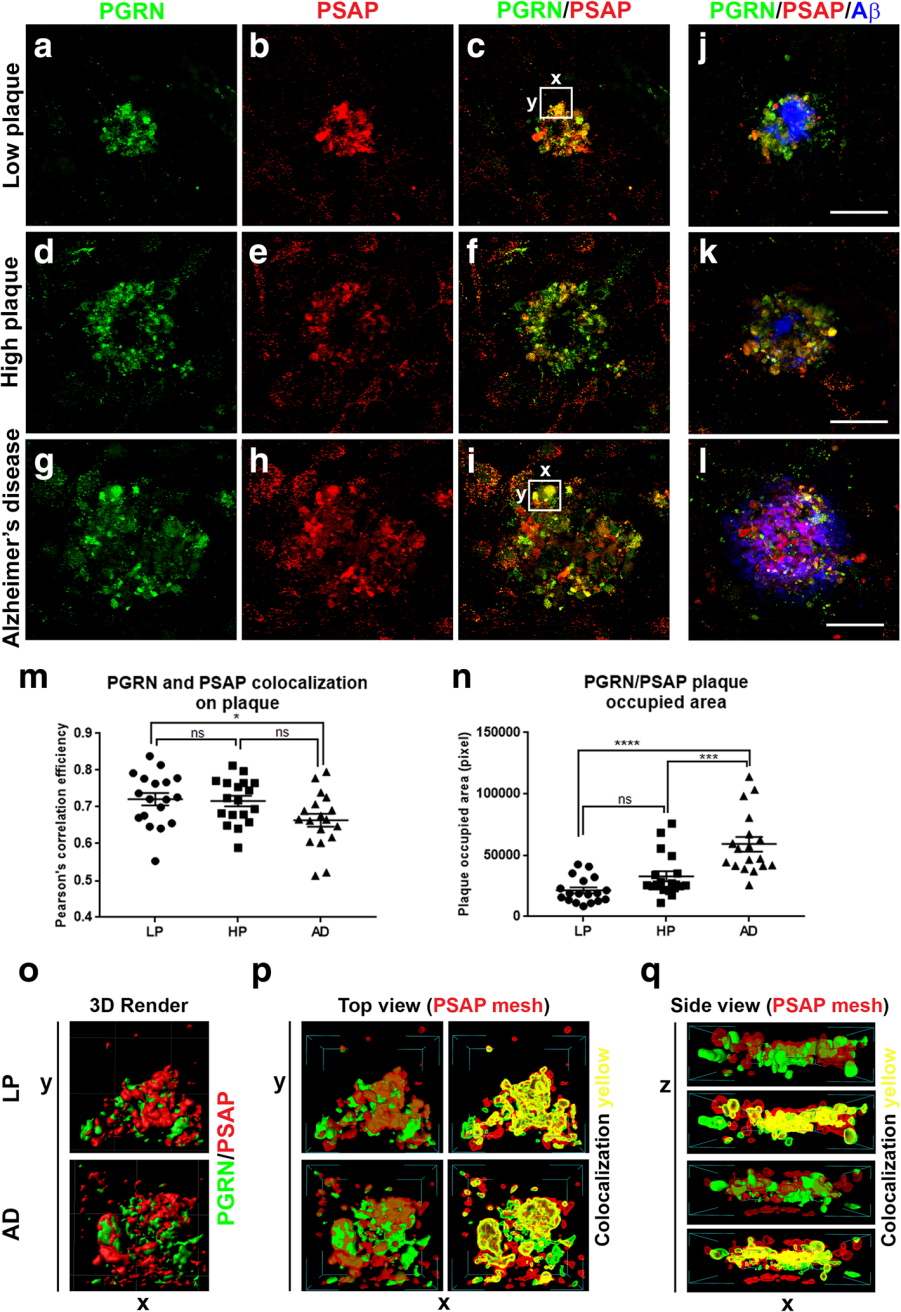
Analysis of PGRN and PSAP colocalization on Aβ plaques in MTG tissue sections. Colocalization of PGRN and PSAP with Aβ in plaques of low plaque, high plaque and Alzheimer’s disease cases. **(a-l).** Images show representative images of relative patterns of immunoreactivity of PGRN (green), PSAP (red) and their colocalization with Aβ (blue) in low plaque (**a-c, j**), high plaque (**d-f, k**) and Alzheimer’s disease (**g-i, l**) cases. The magnification of the images is constant (scale bar represents 30 μm) to show relative sizes of plaques in these disease groups. (**m**). Scatter plot showing relative amounts of colocalization of PGRN and PSAP as measured by Pearson’s correlation efficiency calculated using ExColocalization plug-in of Image J software (*n* = 9 plaques/group). Results show mean values ±S.E.M. Analysis by one-way ANOVA (* p < 0.05, ns; not significant). Value of 1 indicates complete colocalization and value of 0 indicates no colocalization. (**n**). Area measurements of PGRN/PSAP immunoreactive plaques in LP, HP and AD cases. Three cases/disease group and 6 plaques/case were measured. Results show mean values ±S.E.M. Significant increase in area of PGRN/PSAP associated plaques were detected in AD samples compared to HP and LP cases. Three-dimensional Imaging of PGRN-PSAP interactions in plaques. (**o-q**). Three-dimensional reconstruction image of merged PGRN and PSAP immunoreactive plaques in LP and AD cases of areas highlighted in panel C (LP) and K (AD) confocal images (panel O). Software modelling using mesh rendering of section of plaque (white boxes) showing close interactions of PGRN and PSAP in both LP and AD cases (yellow areas). Top view mesh rendering (P) and Side view mesh rendering (Q).

Examination of plaque structures in low plaque, high plaque and AD cases stained for PGRN and PSAP (Fig. 9) showed extensive colocalization in all groups (Fig. 9a-c–LP; Fig. 9d-f-HP; Fig. 9g-i-AD, but with differences between disease groups. All PGRN-associated plaque structures were positive to different extents with PSAP. Measurement of the degree of colocalization of PGRN and PSAP (as Pearson’s correlation efficiency) showed less colocalization in plaques of AD cases compared to LP cases, but not between LP and HP or HP and AD (Fig. 9m). The Pearson’s correlation efficiency values showed a high degree of colocalization of PGRN and PSAP. In order to carry out measurements of PGRN and PSAP colocalization, sections were double-stained for these proteins, but not also for Aβ. This produced images of approximately equal fluorescent intensity; however, to illustrate the arrangement of Aβ in relation to PGRN and PSAP in the different disease groups, parallel sections from the same cases that were triple-stained for PGRN (green), PSAP (red) and Aβ (blue) are also shown (Fig. 9j-LP, Fig. 9k-HP, Fig. 9l-AD). The PGRN- and PSAP-positive structures in Fig. 9 J-L show the relatively larger Aβ plaques in AD cases compared to LP and HP cases. The occupied area of PGRN-PSAP plaques differed significantly between the disease groups. These area measurements were of PGRN-PSAP/Aβ-occupied areas not just Aβ- occupied areas. The same measurements were used to determine the fluorescent intensities of PGRN (Additional file 7: Figure S7A and S7B) and PSAP immunoreactivity (Additional file 7: Figure S7Cand S7D) on plaques in the different disease groups. The total plaque intensities of PGRN and PSAP in AD cases were significantly less than between LP and HP groups (Additional file 7: Figure S7A and S7C), and similar results were obtained when intensity measures were corrected for plaque size (Additional file 7: Figure S7B and S7D).

The confocal images of PGRN and PSAP in plaque structures were further analyzed using computer-assisted three-dimensional imaging to illustrate colocalization using Mesh software imaging. These images showed the Mesh images illustrating the interaction of PGRN and PSAP in an LP and AD case (white boxes areas in Fig. 9c and Fig. 9i). Mesh rendering of 3D-modelled images facilitate the visualization of the interaction of PGRN (green) structures with PSAP (red) structures (Fig. 9p and Fig. 9q). Unlike the rendering of PGRN and Aβ (Fig. 4u), the patterns of PGRN and PSAP interactions suggest close binding of these two proteins. Similar interactions could be demonstrated from rendered images produced from PGRN and PSAP plaque structures from low plaque and high plaque cases (Fig. 9p and Fig. 9q).

## Discussion

The majority of experimental and neuropathological studies of PGRN have focused on the consequences of GRN gene mutations or deletion in rodent models. Mutation in one GRN allele resulting in lower levels of PGRN protein is a cause of frontotemporal lobar degeneration (FTLD) and the accompanying clinical syndrome frontotemporal dementia (FTD) [62]. As PGRN has multiple cellular properties, the mechanisms that cause neurodegeneration have not definitively been identified. PGRN deficiencies in animal models are associated with increased neuroinflammation [17], increased synaptic pruning [21, 63], and dysregulation of lysosomal function [14, 64]. The situation for PGRN in AD is different as increased levels of PGRN in AD brains measured by ELISA has been reported [19], and confirmed in this study by western blot measurements. Another study showed no significant increase in PGRN protein in frontal cortex of AD brains by western blot, but did detect increased levels of PGRN mRNA in these samples [34]. The overall aim of this study was to further investigate using human brain samples some of the features of PGRN interactions with neuropathology identified in mice models of AD, particularly the nature of PGRN associations with Aβ plaques. The common features of PGRN in human AD brains and AD mouse models are PGRN immunoreactive structures associated with Aβ plaques, and cellular expression of PGRN by neurons and microglia [19, 31–34, 65]. From this study, we showed the interaction of PGRN with PSAP, a molecule with similar properties to PGRN, to be a major feature of PGRN-associated with amyloid plaques.

Increased levels of PGRN in AD brains could be considered a reparative feature to prevent further neuropathology as experimental studies have shown that supplementation of PGRN in Grn haploinsufficient mice reduced microglial activation, neuronal lipofuscinosis and improved lysosomal function [25]. Viral transduction of PGRN into an AD transgenic mouse model reduced amyloid accumulation, neuroinflammation and synaptic loss [27], and this treatment protected dopaminergic neurons in a toxin-induced PD mouse model [26]; however, there have been conflicting results from other studies of PGRN in AD mouse models. In one study, reducing PGRN levels resulted in impaired microglial phagocytosis and increased amyloid plaque deposition, while overexpressing PGRN in microglia had the opposite consequence [19]. Using a different AD mouse model (APP/PS1), deficiency of PGRN was associated with reduced deposition of diffuse amyloid due to enhancement of microglial phagocytosis caused by PGRN deficiency increasing expression of microglia TYROBP genes [21]. This study and another observed increased tangle-associated phosphorylated tau with PGRN deficiency in P301L tau mutation mice [66].

In this report, cellular and pathological localization of PGRN was carried out with a well-characterized PGRN antibody raised against a glycosylated recombinant fragment of PGRN corresponding to almost full-length PGRN. This antibody will be able to recognize multiple epitopes of PGRN and appeared to have excellent sensitivity for detection of PGRN in tissue samples. It had been shown to have excellent specificity with no staining or polypeptide bands detected against PGRN knock-out cell samples in contrast to other antibodies [61]. We confirmed its specificity by peptide absorption with immunohistochemistry and western blot analyses. This antibody detected polypeptides of 75–80 kDa in brain samples, the expected size for full-length PGRN. Our studies showed that sensitivity of detection was enhanced in the absence of reducing agents and by fixation of western blot membranes with paraformaldehyde. With this antibody, we demonstrated expression by microglia, neurons and in the cerebrovasculature but not by astrocytes. Staining of structures closely associated with Aβ plaques but not neurofibrillary tangles was observed. In a previous study, the predominant PGRN polypeptide detected using a peptide-derived monoclonal antibody had a molecular weight of approximately 55 kDa, which we showed corresponded to unglycosylated PGRN [34]. This study did not detect the abundant species of PGRN of 75–80 kDa in brain detected in this report. These authors also detected neurofibrillary tangles positive for PGRN, while we observed no significant association of PGRN-immunoreactivity of neurofibrillary tangles identified using two different antibodies to separate epitopes of phosphorylated tau.

A key issue to address in this study is whether the PGRN immunoreactive structures being identified in brain sections are full-length PGRN or proteolytically-processed granulin peptides. Western blot results with the goat antibody to PGRN identified full-length PGRN as the most abundantly present in all brain samples, while lower molecular weight granulin peptides were not readily detectable, or were at very low abundance. This antibody has been shown to recognize granulin peptides if present in samples [61]. A recent study used granulin-domain antibodies to identify immunoreactive structures in AD brains similar to what we have characterized [35]; however the granulin sub-domain antibodies used would have the ability to also detect PGRN, so the issue of the amount of PGRN compared to granulin in plaque structures will require further investigation.

It had been hypothesized that a deficit in PGRN might be an early feature of AD in the prodromal stage [19], and the increase occurred later in the disease as pathology developed. We investigated this using pathologically-staged LP, HP and AD cases. We examined the levels of PGRN protein by western blot, and quantified the numbers and sizes of PGRN-associated amyloid plaques in each of these groups. A limitation to this study was that there were fewer protein extract samples available compared to fixed tissue sections, but all of the cases with available protein extract also had tissue sections. There was no difference in total brain PGRN protein levels between LP and HP cases, but there was a difference between these groups in the number and area of plaques with PGRN-immunoreactive structures. The presence and increase in numbers of PGRN-positive plaques suggest that once Aβ plaques form, PGRN becomes associated with them. In the LP cases, 7 of the cases had no Aβ plaques, while the remaining 9 with plaques all had PGRN-associated with Aβ plaques. In all of the groups, the plaques without PGRN-associated structures tended to have a diffuse morphology and were negative for staining with thioflavin-S, while the plaques with PGRN-associated structures had thioflavin-S positive, aggregated morphologies (Fig. 3d).

We investigated the nature of PGRN structures associated with plaques in terms of their interaction with lysosomal proteins as it had been observed using 5xFAD AD model mice that most PGRN associated with plaques was present within aberrant accumulations of lysosomes [32]. In these mice, most plaques had significant amounts of LAMP-1, CD68 and other lysosomal proteins associated with them, with PGRN and LAMP-1 showing significant colocalization. Our study demonstrated LAMP-1 immunoreactivity associated with plaques but only limited colocalization with PGRN immunoreactivity. CD68 immunoreactivity associated with plaques colocalized with PGRN immunoreactivity, but the majority of plaque-associated PGRN did not colocalize with these lysosomal proteins. The question arising from these observations is whether PGRN associated with plaques was enhancing plaque development, promoting its removal or was not in a biologically active form. On account of our biochemical studies showing that PGRN immunoprecipitated from brain samples pulled down PSAP, the involvement of this protein with PGRN in plaques became an additional feature of this study.

This study has made a number of observations concerning PSAP in relation to AD pathology. With the human brain samples available, we were able to examine PGRN and PSAP changes early in the pathological stages of plaque and tangle formation. Using these samples for co-immunoprecipitation studies of human brain samples, we showed that precipitating PGRN consistently pulled down PSAP but with little difference between the different disease groups. This is the first demonstration of PGRN/PSAP interactions in human brain samples. These PGRN immunoprecipitated samples were negative for sortilin [67, 68]; this finding was unexpected as sortilin is enriched in plaques and regulates PGRN levels [53, 69]. Other proteins that did not interact with PGRN were TMEM106B [34, 70], cathepsin D [71], EphA2 [72] and BACE-1 [32]. Other proteins have been identified to interact with PGRN that were not assessed. These include phospholipase D3 (PLD3) that colocalizes with PGRN on neuritic plaques [73], and Toll-like receptor-9 whose signaling in macrophages is regulated by granulin [74]. However, we could detect PSAP binding to cathepsin D and BACE-1 in brain samples. This will be investigated in further studies. The possible role of PSAP in AD has not been adequately addressed. There were no changes in microglial or neuronal PSAP immunoreactivity in AD sections compared to those from FTLD cases due to GRN mutations where neuronal PSAP was reduced, but microglia and astrocyte expression was increased [42]. Our findings from this study were increased levels of PSAP in AD brains, with significant positive correlation between PGRN and PSAP levels in all samples. We confirmed PGRN and PSAP interactions in neurons and microglia, but most significantly might be the colocalization of PGRN and PSAP associated with plaque structures. Increased levels of PSAP have been shown to increase the oligomerization of PGRN [42]. The interaction of PGRN and PSAP into aggregated structures may result in loss of biological activities, a feature that will need to be investigated. Even though there are increased amounts of PGRN and/or PSAP around plaques, being bound into these structures may prevent cellular signaling needed for their protective properties. It had been hypothesized that increased PGRN should stimulate associated microglia to phagocytose and remove the plaques, however if the PGRN is in a form that does not permit cellular endocytosis, namely bound with PSAP, excess amounts of inactive PGRN protein might be hindering plaque removal or promoting Aβ deposition. We observed that PGRN/PSAP-positive Aβ plaques in AD cases appeared to extend beyond the zones of PGRN/PSAP deposits. The biological properties of PGRN bound with PSAP in extracellular locations have not been investigated.

The significance of PSAP in neurodegenerative diseases is just being appreciated. A recent study employing new proteomics methods identified PSAP as a CSF biomarker for distinguishing preclinical AD from AD [44]. As PGRN has been studied as a biomarker for CSF and plasma but with limited diagnostic utility [30], improved diagnostic results might occur by combining both of these factors in biomarker discovery studies. We found most PSAP expression in brain samples was in neurons, which strongly colocalized with PGRN. PSAP expression by microglia and astrocytes was very limited in AD and aged brains but we observed that most PGRN in plaques colocalized with PSAP. Induction of microglial and astrocytic PSAP expression was reported in acute injury. Using the acute cortical stab wound model in mice, a 10-fold increase in PGRN was detected and a 50% increase in PSAP [42]. Experimental studies have shown that PSAP can regulate the levels and aggregation state of PGRN. Reducing levels of PSAP resulted in increased levels of PGRN in vitro, and PSAP gene-deficient mice had higher levels of PGRN. However, PSAP overexpression also induced increased amounts of aggregated PGRN, but not GRN mRNA [39]. One of the initial questions about PGRN in Aβ plaques was whether the presence of this factor affected the aggregation and/or removal of the plaques. In vitro experiments have shown that PGRN can stimulate microglial phagocytosis but the PGRN associated with  plaques, which is detectable in sections from non-AD low plaque cases, does not appear to promote the removal of plaques. PGRN/PSAP plaques are infiltrated by microglia but these cells do not appear capable of removing Aβ. Our results seem to indicate that Aβ deposition increases in AD brains irrespective of the presence of PGRN and PSAP.

In summary, we have described features of PGRN and PSAP in a staged series of human brain tissue samples, and in particular PGRN-positive plaques. The most noticeable feature was the interactions in plaques of PGRN with PSAP. It is possible that PGRN/PSAP interactions with plaques could result in sequestration of toxic forms of Aβ, however, to address this as a mechanism, further studies will determine whether PGRN interactions with PSAP affect its neuroprotective and anti-inflammatory properties. This could be an important issue when determining whether PGRN supplementation will be useful. If the excess PGRN protein becomes absorbed by plaques in AD cases, it might not have the expected neuroprotective properties. The possible role for PSAP supplementation can also be considered. Both PGRN and PSAP have similar growth factor and lysosomal regulatory functions, but the consequences of PSAP supplementation has not been explored.

## Conclusion

From these findings, it can be concluded that the presence of neuroprotective molecules PGRN and PSAP on Aβ plaques do not prevent the formation of these structures. Significant interactions between these molecules were demonstrated biochemically, and by immunohistochemical techniques in neurons and also associated with Aβ plaques. Secreted forms of PGRN have multiple protective and anti-inflammatory properties, but these might not be evident when this protein is deposited with PSAP around Aβ plaques. Increased total protein levels of PGRN and PSAP was evident in AD samples but not those with less pathology, but the presence of PGRN and PSAP on most plaques in low pathology control cases with fewer smaller plaques indicate this is an earlier event in development of AD pathology. Binding of these proteins on Aβ plaques might have ameliorating effects of Aβ toxicity on surrounding cells, or it might be hindering the removal of Aβ plaque by infiltrated microglia.

## Supplementary information

**Additional file 1 Figure S1**. (**A**-**B**). PGRN peptide absorption of goat anti-PGRN antibody (AF2420**).** Low magnification images demonstrating absence of immunoreactive structures in MTG (AD case) reacted with PGRN antibody preabsorbed with PGRN peptide (**A**)(+PGRN peptide) and demonstration of significant immunoreactivity in parallel section from same case reacted with non-peptide antibody (**B**)(−PGRN peptide). Scale bars represent 200 μm. (**C**-**D**). PSAP peptide absorption of rabbit anti-PSAP antibody (AF8470). Low magnification images demonstrating absence of immunoreactive structures in MTG (AD case) reacted with PSAP antibody preabsorbed with PSAP peptide (**C**)(+PSAP peptide) and demonstration of significant immunoreactivity in parallel section from same case reacted with non-peptide antibody (**D**)(−PSAP peptide). Scale bars represent 200 μm.

**Additional file 2 Figure S2.** Western blot characterization of PGRN polypeptides detected with goat anti-PGRN antibody (AF2420). **A**). Absorption of PGRN polypeptides with peptide-absorbed PGRN antibody. Western blot images of purified PGRN peptide (PGRN), AD brain protein extract (AD) and THP macrophage cell protein extracts (THP) detected with PGRN peptide-absorbed antibody (+Peptide) or non-absorbed antibody (−Peptide). Peptide absorption resulted in almost complete absence of PGRN polypeptide bands. **B**). Detection of PGRN by western blots is sensitive to reducing agents. Western blots comparing polypeptide bands in protein extracts of samples prepared with DTT (+) or without DTT (−) as reducing agent. Samples: PGRN; purified recombinant PGRN protein. LAN-5; neuronal cells. THP; THP-1 derived macrophages. Brain; AD brain samples. Blots were probed with goat anti-PGRN antibody (R&D Systems, AF2420:50 ng/ml). C). Sensitivity of detection of PGRN polypeptides in brain samples is enhanced by membrane fixation with paraformaldehyde vapors. Western blot images of brain protein samples from low plaque (LP) and Alzheimer’s disease (AD) cases separated under identical conditions without reducing agents. One membrane was fixed with paraformaldehyde vapors (+PFA) compared to membrane not PFA treated (−PFA). Sensitivity of detection is enhanced in PFA fixed membranes. Blots were probed with goat anti-PGRN antibody (R&D Systems, AF2420:50 ng/ml). D). Identification of deglycosylated forms of PGRN. Protein extracts from THP macrophage cells (THP), and LP and AD brain samples were treated with deglycosylation enzyme PNGaseF (+) or control treated (−). Deglycosylation treatment resulted in increased levels of 55 kDa polypeptides and reduced amounts of ~ 75 kDa PGRN band. Blots were probed with goat anti-PGRN antibody (R&D Systems, AF2420:50 ng/ml).

**Additional file 3 Figure S3** Limited colocalization of PGRN with markers defining neurites and neuritic plaques. **A**-**C**): Confocal image of PGRN (green)(**A**) immunoreactivity associated with a neuritic plaque identified with pTau antibody AT180 (B) with merged image (**C**) in an AD case showing no colocalization of staining. DAPI staining identified nuclei and also highly aggregated amyloid within plaques. Scale bar represents 20 μm. **D**-**F**): Confocal image of PGRN (green)(D) immunoreactivity associated with neurites and neuritic plaque identified with pan-neurofilament antibody SMI312 (**E**) with merged DAPI-stained image (**F**) in an AD case showing limited colocalization of staining. Scale bar represents 20 μm. G-I): Confocal image of PGRN (green)(**G**) immunoreactivity associated with neurites and neuritic plaque identified with synaptophysin antibody (**H**) with merged DAPI-stained image (**I**) in an AD case showing no colocalization of staining. Scale bar represents 20 μm. These images were acquired using a Leica SP8 confocal microscope.

**Additional file 4 Figure S4.** Complete western blot images of MTG protein samples. A). Complete western blot images of MTG brain sauremples probed with antibody to detect PGRN polypeptides in Low plaque (LP), high plaque (HP) and AD samples. Major PGRN polypeptide bands detected at approximately 75–80 kDa, with less intense bands at 55 kDa. These blots showed absence of low molecular weight granulin peptides. *Top Image*: Shorter exposure of western blot. *Lower Image*: Longer exposure of western blot to demonstrate if granulin peptides were present. **B**). Complete western blot images of MTG brain samples probed with antibody to detect PSAP polypeptides in Low plaque (LP), high plaque (HP) and AD samples. Major PSAP polypeptide bands detected at approximately 72 kDa. Additional polypeptide bands are detectable with image enhancement indicating detection of PSAP-derived saposin peptides. *Top Image*: Shorter exposure of western blot. *Lower image*: Longer exposure of western blot images of MTG brain samples probed with antibody to detect PSAP polypeptides. Bands with molecular weights of saposin peptides are indicated. These were noticeable in AD samples.

**Additional file 5 Figure S5.** Additional analysis and controls for proteins analyzed by co-immunoprecipitation or colocalization with progranulin and prosaposin A-B) Composite western blot of additional potential PGRN-interacting proteins using Protein A or Protein G beads conjugated with normal goat IgG, or antibodies to PGRN, PSAP, sortilin, TMEM106B, cathepsin D and beta secretase-1 (BACE-1) (panel B). Comparison was carried out with non-immunoprecipitated total protein extracts of brain samples analyzed on the same blots. Sortilin: Antibody detected polypeptide with expected molecular weight in brain samples. No sortilin immunoreactive bands were present in PGRN IP samples but a faint band was detected in PSAP IP samples. TMEM106B: Antibody detected polypeptide with expected molecular weight in brain samples. No TMEM106B immunoreactive bands were present in PGRN or PSAP immunoprecipitated samples. Cathepsin D: Antibody detected polypeptide with expected molecular weight in brain samples. No cathepsin D immunoreactive bands were present in PGRN IP samples but a faint band was detected in PSAP IP samples. BACE1: Analysis showed that samples precipitated with PGRN conjugated beads did not pull down BACE1 immunoreactive bands but band detected in PSAP-immunoprecipitated samples. BACE-1 conjugated beads included as positive control. (SH – extract of neuronal SH-SY5Y cells). C) Scatter blot showing ratio of expression levels of PSAP to amounts of PGRN in immunoprecipitated samples. The ratio of PSAP to PGRN present in samples immunoprecipitated with antibody to PGRN was not significantly different between disease groups. Confocal microscopy of PGRN-positive plaque structures with indicated antibodies. D-F) PGRN (green) and Sortilin (red) immunoreactivity was detected in plaques but there was no detectable colocalization (F). Scale bars represent 30 μm. G-I) PGRN (green) and TMEM106B (red) immunoreactivity was detected in or around plaques but there was no detectable colocalization (I). Scale bars represent 30 μm. J-L) PGRN (green) and Cathepsin D (red) immunoreactivity. There was no detectable colocalization (L). Scale bars represent 30 μm. M-O) PGRN (green) and BACE (red) immunoreactivity was detected on cells around plaques but there was no detectable colocalization in cells and on plaques (O). Scale bars represent 30 μm.

**Additional file 6 Figure S6** Complete western blot images of immunoprecipitated brain and cell samples to identify presence of granulin or saposin peptides. **A**) Complete western blot image of brain samples immunoprecipitated with PGRN antibody conjugated magnetic beads and detected with antibody to PSAP. The blots showed no evidence of lower molecular weight saposin peptides. **B**) Complete western blot image of brain samples immunoprecipitated with PGRN antibody conjugated magnetic beads and detected with antibody to PGRN. The blots showed no evidence of lower molecular weight granulin peptides. **C)** Complete western blot images of PGRN-overexpressing HEK and brain samples (AD) immunoprecipitated with magnetic beads conjugated with control normal goat immunoglobulin (goat IgG), or PGRN or PSAP antibodies and probed with antibody to PGRN. Image shows that granulin peptides could be minimally detected only in HEK cells expressing high levels of PGRN protein. Blot also shows total extracts from SH-SY5Y differentiated neurons (SY) and AD brain analyzed in parallel showing no detectable granulin peptides.

**Additional file 7 Figure S7**: Fluorescent plaque intensities of PGRN and PSAP in MTG sections between disease groups. A) Fluorescent intensity of PGRN immunoreactivity on plaques between LP, HP and AD cases. Six plaques were measured in 3 cases in each group (*n* = 18 plaques/group). Results represent mean + SEM of total fluorescent intensity. Results analyzed by one-way ANOVA with Neuman-Keuls post-hoc test between groups. ** *p* < 0.01, ns: not significant. B) Area-adjusted fluorescent intensity of PGRN immunoreactivity on plaques between LP, HP and AD cases of PGRN-PSAP positive plaques. Six plaques were measured in 3 cases in each group (n = 18 plaques/group). Results represent mean ± S.E.M. of average fluorescent intensity adjusted for plaque area (Fig. 9N). Results analyzed by one-way ANOVA with Neuman-Keuls post hoc test between groups. **** *p* < 0.0001, ** p < 0.01, ns: not significant. C) Fluorescent intensity of PSAP immunoreactivity on plaques between LP, HP and AD cases. Six plaques were measured in 3 cases in each group (n = 18 plaques/group). Results represent mean ±S.E.M. of total fluorescent intensity. Results analyzed by one-way ANOVA with Neuman-Keuls post hoc test between groups. **** p < 0.0001, ns: not significant. D) Area-adjusted fluorescent intensity of PSAP immunoreactivity on plaques between LP, HP and AD cases of PGRN-PSAP positive plaques. Results represent mean + ±S.E.M. of average fluorescent intensity adjusted for plaque area measures (Fig. 9N). Six plaques were measured in 3 cases in each group (n = 18 plaques/group). Results analyzed by one-way ANOVA with Neuman-Keuls post-hoc test between groups. **** p < 0.0001, * *p* < 0.05, ns: not significant.

## References

[1] (2016) World Alzheimer Report 2016. http://www.alz.co.uk/research/WorldAlzheimerReport2016.pdf

[2] DeTure MA, Dickson DW (2019). The neuropathological diagnosis of Alzheimer’s disease. Mol Neurodegener.

[3] Mo J-J, Li J-Y, Yang Z, Liu Z, Feng J-S (2017). Efficacy and safety of anti-amyloid-beta immunotherapy for Alzheimer’s disease: a systematic review and network meta-analysis. Ann Clin Transl Neurol.

[4] Piton M, Hirtz C, Desmetz C, Milhau J, Lajoix AD, Bennys K, Lehmann S, Gabelle A (2018). Alzheimer’s disease: advances in drug development. J Alzheimers Dis.

[5] Sevigny J, Chiao P, Bussière T, Weinreb PH, Williams L, Maier M, Dunstan R, Salloway S, Chen T, Ling Y, O’Gorman J, Qian F (2016). The antibody aducanumab reduces Aβ plaques in Alzheimer’s disease. Nature.

[6] Paushter Daniel H., Du Huan, Feng Tuancheng, Hu Fenghua (2018). The lysosomal function of progranulin, a guardian against neurodegeneration. Acta Neuropathologica.

[7] Daniel R, He Z, Carmichael KP, Halper J, Bateman A (2000). Cellular localization of gene expression for progranulin. J Histochem Cytochem.

[8] Bateman A, Belcourt D, Bennett H, Lazure C, Solomon S (1990). Granulins, a novel class of peptide from leukocytes. Biochem Biophys Res Commun.

[9] Bossu P, Salani F, Alberici A, Archetti S, Bellelli G, Galimberti D, Scarpini E, Spalletta G, Caltagirone C, Padovani A, Borroni B (2011). Loss of function mutations in the progranulin gene are related to pro-inflammatory cytokine dysregulation in frontotemporal lobar degeneration patients. J Neuroinflammation.

[10] Martens LH, Zhang J, Barmada SJ, Zhou P, Kamiya S, Sun B, Min S-W, Gan L, Finkbeiner S, Huang EJ, Farese RVJ (2012). Progranulin deficiency promotes neuroinflammation and neuron loss following toxin-induced injury. J Clin Invest.

[11] Van Damme P, Van Hoecke A, Lambrechts D, Vanacker P, Bogaert E, van Swieten J, Carmeliet P, Van Den Bosch L, Robberecht W (2008). Progranulin functions as a neurotrophic factor to regulate neurite outgrowth and enhance neuronal survival. J Cell Biol.

[12] Gass J, Lee WC, Cook C, Finch N, Stetler C, Jansen-West K, Lewis J, Link CD, Rademakers R, Nykjaer A, Petrucelli L (2012). Progranulin regulates neuronal outgrowth independent of sortilin. Mol Neurodegener.

[13] Tanaka Y, Matsuwaki T, Yamanouchi K, Nishihara M (2013). Increased lysosomal biogenesis in activated microglia and exacerbated neuronal damage after traumatic brain injury in progranulin-deficient mice. Neuroscience.

[14] Tanaka Y, Suzuki G, Matsuwaki T, Hosokawa M, Serrano G, Beach TG, Yamanouchi K, Hasegawa M, Nishihara M (2017). Progranulin regulates lysosomal function and biogenesis through acidification of lysosomes. Hum Mol Genet.

[15] Baker M, Mackenzie IR, Pickering-Brown SM, Gass J, Rademakers R, Lindholm C, Snowden J, Adamson J, Sadovnick AD, Rollinson S, Cannon A, Dwosh E (2006). Mutations in progranulin cause tau-negative frontotemporal dementia linked to chromosome 17. Nature.

[16] Mackenzie IRA (2007). The neuropathology and clinical phenotype of FTD with progranulin mutations. Acta Neuropathol.

[17] Ma Y, Matsuwaki T, Yamanouchi K, Nishihara M (2017). Involvement of progranulin in modulating neuroinflammatory responses but not neurogenesis in the hippocampus of aged mice. Exp Gerontol.

[18] Arrant AE, Filiano AJ, Patel AR, Hoffmann MQ, Boyle NR, Kashyap SN, Onyilo VC, Young AH, Roberson ED (2018). Reduction of microglial progranulin does not exacerbate pathology or behavioral deficits in neuronal progranulin-insufficient mice. Neurobiol Dis.

[19] Minami SS, Min S-W, Krabbe G, Wang C, Zhou Y, Asgarov R, Li Y, Martens LH, Elia LP, Ward ME, Mucke L, Farese RVJ, Gan L (2014). Progranulin protects against amyloid beta deposition and toxicity in Alzheimer’s disease mouse models. Nat Med.

[20] Roberson ED, Filiano AJ, Martens LH, Young AH, Warmus BA, Zhou P, Diaz-Ramirez G, Jiao J, Zhang Z, Huang EJ, Gao FB, Farese RV (2013). Dissociation of frontotemporal dementia-related deficits and neuroinflammation in progranulin haploinsufficient mice. Ann Intern Med.

[21] Takahashi H, Klein ZA, Bhagat SM, Kaufman AC, Kostylev MA, Ikezu T, Strittmatter SM (2017). Opposing effects of progranulin deficiency on amyloid and tau pathologies via microglial TYROBP network. Acta Neuropathol.

[22] Yin F, Dumont M, Banerjee R, Ma Y, Li H, Lin MT, Beal MF, Nathan C, Thomas B, Ding A (2010). Behavioral deficits and progressive neuropathology in progranulin-deficient mice: a mouse model of frontotemporal dementia. FASEB J.

[23] Arrant AE, Filiano AJ, Unger DE, Young AH, Roberson ED (2017). Restoring neuronal progranulin reverses deficits in a mouse model of frontotemporal dementia. Brain.

[24] Ward ME, Chen R, Huang H-Y, Ludwig C, Telpoukhovskaia M, Taubes A, Boudin H, Minami SS, Reichert M, Albrecht P, Gelfand JM, Cruz-Herranz A (2012). Possible involvement of lysosomal dysfunction in pathological changes of the brain in aged progranulin-deficient mice. Acta Neuropathol.

[25] Arrant AE, Onyilo VC, Unger DE, Roberson ED (2018). Progranulin gene therapy improves Lysosomal dysfunction and microglial pathology associated with Frontotemporal dementia and neuronal Ceroid Lipofuscinosis. J Neurosci.

[26] Van Kampen Jackalina M., Baranowski David, Kay Denis G. (2014). Progranulin Gene Delivery Protects Dopaminergic Neurons in a Mouse Model of Parkinson’s Disease. PLoS ONE.

[27] Van Kampen Jackalina M., Kay Denis G. (2017). Progranulin gene delivery reduces plaque burden and synaptic atrophy in a mouse model of Alzheimer's disease. PLOS ONE.

[28] Kamalainen A, Viswanathan J, Natunen T, Helisalmi S, Kauppinen T, Pikkarainen M, Pursiheimo J-P, Alafuzoff I, Kivipelto M, Haapasalo A, Soininen H, Herukka S-K, Hiltunen M (2013). GRN variant rs5848 reduces plasma and brain levels of granulin in Alzheimer’s disease patients. J Alzheimers Dis.

[29] Morenas-Rodriguez E, Cervera-Carles L, Vilaplana E, Alcolea D, Carmona-Iragui M, Dols-Icardo O, Ribosa-Nogue R, Munoz-Llahuna L, Sala I, Belen Sanchez-Saudinos M, Blesa R, Clarimon J (2016). Progranulin protein levels in cerebrospinal fluid in primary neurodegenerative dementias. J Alzheimers Dis.

[30] Suárez‐Calvet Marc, Capell Anja, Araque Caballero Miguel Ángel, Morenas‐Rodríguez Estrella, Fellerer Katrin, Franzmeier Nicolai, Kleinberger Gernot, Eren Erden, Deming Yuetiva, Piccio Laura, Karch Celeste M, Cruchaga Carlos, Paumier Katrina, Bateman Randall J, Fagan Anne M, Morris John C, Levin Johannes, Danek Adrian, Jucker Mathias, Masters Colin L, Rossor Martin N, Ringman John M, Shaw Leslie M, Trojanowski John Q, Weiner Michael, Ewers Michael, Haass Christian (2018). CSF progranulin increases in the course of Alzheimer's disease and is associated with sTREM2, neurodegeneration and cognitive decline. EMBO Molecular Medicine.

[31] Gliebus G, Rosso A, Lippa CF (2009). Progranulin and beta-amyloid distribution: a case report of the brain from preclinical PS-1 mutation carrier. Am J Alzheimers Dis Other Dement.

[32] Gowrishankar S, Yuan P, Wu Y, Schrag M, Paradise S, Grutzendler J, De Camilli P, Ferguson SM (2015). Massive accumulation of luminal protease-deficient axonal lysosomes at Alzheimer’s disease amyloid plaques. Proc Natl Acad Sci U S A.

[33] Pereson S, Wils H, Kleinberger G, McGowan E, Vandewoestyne M, Van Broeck B, Joris G, Cuijt I, Deforce D, Hutton M, Van Broeckhoven C, Kumar-Singh S (2009). Progranulin expression correlates with dense-core amyloid plaque burden in Alzheimer disease mouse models. J Pathol.

[34] Satoh J-I, Kino Y, Kawana N, Yamamoto Y, Ishida T, Saito Y, Arima K (2014). TMEM106B expression is reduced in Alzheimer’s disease brains. Alzheimers Res Ther.

[35] Mao Q, Wang D, Li Y, Kohler M, Wilson J, Parton Z, Shmaltsuyeva B, Gursel D, Rademakers R, Weintraub S, Mesulam MM, Xia H, Bigio EH (2017). Disease and region specificity of granulin immunopositivities in Alzheimer disease and frontotemporal lobar degeneration. J Neuropathol Exp Neurol.

[36] Liu B, Mosienko V, Vaccari Cardoso B, Prokudina D, Huentelman M, Teschemacher AG, Kasparov S (2018). Glio- and neuro-protection by prosaposin is mediated by orphan G-protein coupled receptors GPR37L1 and GPR37. Glia.

[37] Meyer RC, Giddens MM, Coleman BM, Hall RA (2014). The protective role of prosaposin and its receptors in the nervous system. Brain Res.

[38] Nabeka H, Saito S, Li X, Shimokawa T, Khan MSI, Yamamiya K, Kawabe S, Doihara T, Hamada F, Kobayashi N, Matsuda S (2017). Interneurons secrete prosaposin, a neurotrophic factor, to attenuate kainic acid-induced neurotoxicity. IBRO reports.

[39] Nicholson AM, Finch NA, Almeida M, Perkerson RB, van Blitterswijk M, Wojtas A, Cenik B, Rotondo S, Inskeep V, Almasy L, Dyer T, Peralta J (2016). Prosaposin is a regulator of progranulin levels and oligomerization. Nat Commun.

[40] Zhou X, Sullivan PM, Sun L, Hu F (2017). The interaction between progranulin and prosaposin is mediated by granulins and the linker region between saposin B and C. J Neurochem.

[41] Zhou X, Sun L, Bastos de Oliveira F, Qi X, Brown WJ, Smolka MB, Sun Y, Hu F (2015). Prosaposin facilitates sortilin-independent lysosomal trafficking of progranulin. J Cell Biol.

[42] Zhou X, Sun L, Bracko O, Choi JW, Jia Y, Nana AL, Brady OA, Hernandez JCC, Nishimura N, Seeley WW, Hu F (2017). Impaired prosaposin lysosomal trafficking in frontotemporal lobar degeneration due to progranulin mutations. Nat Commun.

[43] Nabeka H, Uematsu K, Takechi H, Shimokawa T, Yamamiya K, Li C, Doihara T, Saito S, Kobayashi N, Matsuda S (2014). Prosaposin overexpression following kainic acid-induced neurotoxicity. PLoS One.

[44] Andersson A, Remnestal J, Nellgard B, Vunk H, Kotol D, Edfors F, Uhlen M, Schwenk JM, Ilag LL, Zetterberg H, Blennow K, Manberg A (2019). Development of parallel reaction monitoring assays for cerebrospinal fluid proteins associated with Alzheimer’s disease. Clin Chim Acta.

[45] Beach TG, Adler CH, Sue LI, Serrano G, Shill HA, Walker DG, Lue L, Roher AE, Dugger BN, Maarouf C, Birdsill AC, Intorcia A (2015). Arizona study of aging and neurodegenerative disorders and brain and body donation program. Neuropathology.

[46] McKeith IG, Dickson DW, Lowe J, Emre M, O’Brien JT, Feldman H, Cummings J, Duda JE, Lippa C, Perry EK, Aarsland D, Arai H (2005). Diagnosis and management of dementia with Lewy bodies: third report of the DLB consortium. Neurology.

[47] Newell KL, Hyman BT, Growdon JH, Hedley-Whyte ET (1999). Application of the National Institute on Aging NIA-Reagan institute criteria for the neuropathological diagnosis of Alzheimer disease. J Neuropathol Exp Neurol.

[48] Beach TG, Sue LI, Walker DG, Sabbagh MN, Serrano G, Dugger BN, Mariner M, Yantos K, Henry-Watson J, Chiarolanza G, Hidalgo JA, Souders L (2012). Striatal amyloid plaque density predicts Braak neurofibrillary stage and clinicopathological Alzheimer’s disease: implications for amyloid imaging. J Alzheimers Dis.

[49] Beach TG, Adler CH, Lue L, Sue LI, Bachalakuri J, Henry-Watson J, Sasse J, Boyer S, Shirohi S, Brooks R, Eschbacher J, White CL (2009). Unified staging system for Lewy body disorders: correlation with nigrostriatal degeneration, cognitive impairment and motor dysfunction. Acta Neuropathol.

[50] Hixson JE, Vernier DT (1990). Restriction isotyping of human apolipoprotein E by gene amplification and cleavage with HhaI. J Lipid Res.

[51] Walker DG, Tang TM, Lue L-F (2018). Increased expression of toll-like receptor 3, an anti-viral signaling molecule, and related genes in Alzheimer’s disease brains. Exp Neurol.

[52] Walker DG, Whetzel AM, Serrano G, Sue LI, Beach TG, Lue LF (2015). Association of CD33 polymorphism rs3865444 with Alzheimer’s disease pathology and CD33 expression in human cerebral cortex. Neurobiol Aging.

[53] Hu X, Hu ZL, Li Z, Ruan CS, Qiu WY, Pan A, Li CQ, Cai Y, Shen L, Chu Y, Tang BS, Cai H et al (2017) Sortilin fragments deposit at senile plaques in human cerebrum. Front Neuroanat 11:45. 10.3389/fnana.2017.00045 10.3389/fnana.2017.00045 PMC5461299 28638323

[54] Walker, Lue, Beach, Tooyama (2019). Microglial Phenotyping in Neurodegenerative Disease Brains: Identification of Reactive Microglia with an Antibody to Variant of CD105/Endoglin. Cells.

[55] Schneider CA, Rasband WS, Eliceiri KW (2012). NIH image to ImageJ: 25 years of image analysis. Nat Methods.

[56] Stauffer W, Sheng H, Lim HN (2018). EzColocalization: an ImageJ plugin for visualizing and measuring colocalization in cells and organisms. Sci Rep.

[57] Lee BR, Kamitani T (2011). Improved immunodetection of endogenous alpha-synuclein. PLoS One.

[58] Preterre C, Corbille A-G, Balloy G, Letournel F, Neunlist M, Derkinderen P (2015). Optimizing Western blots for the detection of endogenous alpha-Synuclein in the enteric nervous system. J Park Dis.

[59] Sasaki A, Arawaka S, Sato H, Kato T (2015). Sensitive western blotting for detection of endogenous Ser129-phosphorylated alpha-synuclein in intracellular and extracellular spaces. Sci Rep.

[60] Amatruda TT, Sidell N, Ranyard J, Koeffler HP (1985). Retinoic acid treatment of human neuroblastoma cells is associated with decreased N-myc expression. Biochem Biophys Res Commun.

[61] Holler Christopher J., Taylor Georgia, Deng Qiudong, Kukar Thomas (2017). Intracellular Proteolysis of Progranulin Generates Stable, Lysosomal Granulins that Are Haploinsufficient in Patients with Frontotemporal Dementia Caused by GRN Mutations. eneuro.

[62] Mackenzie IRA, Baker M, Pickering-Brown S, Hsiung G-YR, Lindholm C, Dwosh E, Gass J, Cannon A, Rademakers R, Hutton M, Feldman HH (2006). The neuropathology of frontotemporal lobar degeneration caused by mutations in the progranulin gene. Brain.

[63] Lui Hansen, Zhang Jiasheng, Makinson Stefanie R., Cahill Michelle K., Kelley Kevin W., Huang Hsin-Yi, Shang Yulei, Oldham Michael C., Martens Lauren Herl, Gao Fuying, Coppola Giovanni, Sloan Steven A., Hsieh Christine L., Kim Charles C., Bigio Eileen H., Weintraub Sandra, Mesulam Marek-Marsel, Rademakers Rosa, Mackenzie Ian R., Seeley William W., Karydas Anna, Miller Bruce L., Borroni Barbara, Ghidoni Roberta, Farese Robert V., Paz Jeanne T., Barres Ben A., Huang Eric J. (2016). Progranulin Deficiency Promotes Circuit-Specific Synaptic Pruning by Microglia via Complement Activation. Cell.

[64] Gotzl JK, Colombo A-V, Fellerer K, Reifschneider A, Werner G, Tahirovic S, Haass C, Capell A (2018). Early lysosomal maturation deficits in microglia triggers enhanced lysosomal activity in other brain cells of progranulin knockout mice. Mol Neurodegener.

[65] Ahmed Z, Mackenzie IRA, Hutton ML, Dickson DW (2007). Progranulin in frontotemporal lobar degeneration and neuroinflammation. J Neuroinflammation.

[66] Hosokawa M, Arai T, Masuda-Suzukake M, Kondo H, Matsuwaki T, Nishihara M, Hasegawa M, Akiyama H (2015). Progranulin reduction is associated with increased tau phosphorylation in P301L tau transgenic mice. J Neuropathol Exp Neurol.

[67] Hu F, Padukkavidana T, Vaegter CB, Brady OA, Zheng Y, Mackenzie IR, Feldman HH, Nykjaer A, Strittmatter SM (2010). Sortilin-mediated endocytosis determines levels of the frontotemporal dementia protein, progranulin. Neuron.

[68] Zheng Yanqiu, Brady Owen A., Meng Peter S., Mao Yuxin, Hu Fenghua (2011). C-Terminus of Progranulin Interacts with the Beta-Propeller Region of Sortilin to Regulate Progranulin Trafficking. PLoS ONE.

[69] Zhou F-Q, Jiang J, Griffith CM, Patrylo PR, Cai H, Chu Y, Yan X-X (2018). Lack of human-like extracellular sortilin neuropathology in transgenic Alzheimer’s disease model mice and macaques. Alzheimers Res Ther.

[70] Finch N, Carrasquillo MM, Baker M, Rutherford NJ, Coppola G, Dejesus-Hernandez M, Crook R, Hunter T, Ghidoni R, Benussi L, Crook J, Finger E (2011). TMEM106B regulates progranulin levels and the penetrance of FTLD in GRN mutation carriers. Neurology.

[71] Beel S, Moisse M, Damme M, De Muynck L, Robberecht W, Van Den Bosch L, Saftig P, Van Damme P (2017). Progranulin functions as a cathepsin D chaperone to stimulate axonal outgrowth in vivo. Hum Mol Genet.

[72] Neill T, Buraschi S, Goyal A, Sharpe C, Natkanski E, Schaefer L, Morrione A, Iozzo RV (2016). EphA2 is a functional receptor for the growth factor progranulin. J Cell Biol.

[73] Satoh J-I, Kino Y, Yamamoto Y, Kawana N, Ishida T, Saito Y, Arima K (2014). PLD3 is accumulated on neuritic plaques in Alzheimer’s disease brains. Alzheimers Res Ther.

[74] Park B, Buti L, Lee S, Matsuwaki T, Spooner E, Brinkmann MM, Nishihara M, Ploegh HL (2011). Granulin is a soluble cofactor for toll-like receptor 9 signaling. Immunity.

