# Supplementary figures and images for: Characterization of lysosomal proteins Progranulin and Prosaposin and their interactions in Alzheimer’s disease and aged brains: increased levels correlate with neuropathology
Source: Acta Neuropathol Commun. 2019 Dec 21;7:215. doi: 10.1186/s40478-019-0862-8 (PMC6925443; doi:10.1186/s40478-019-0862-8)

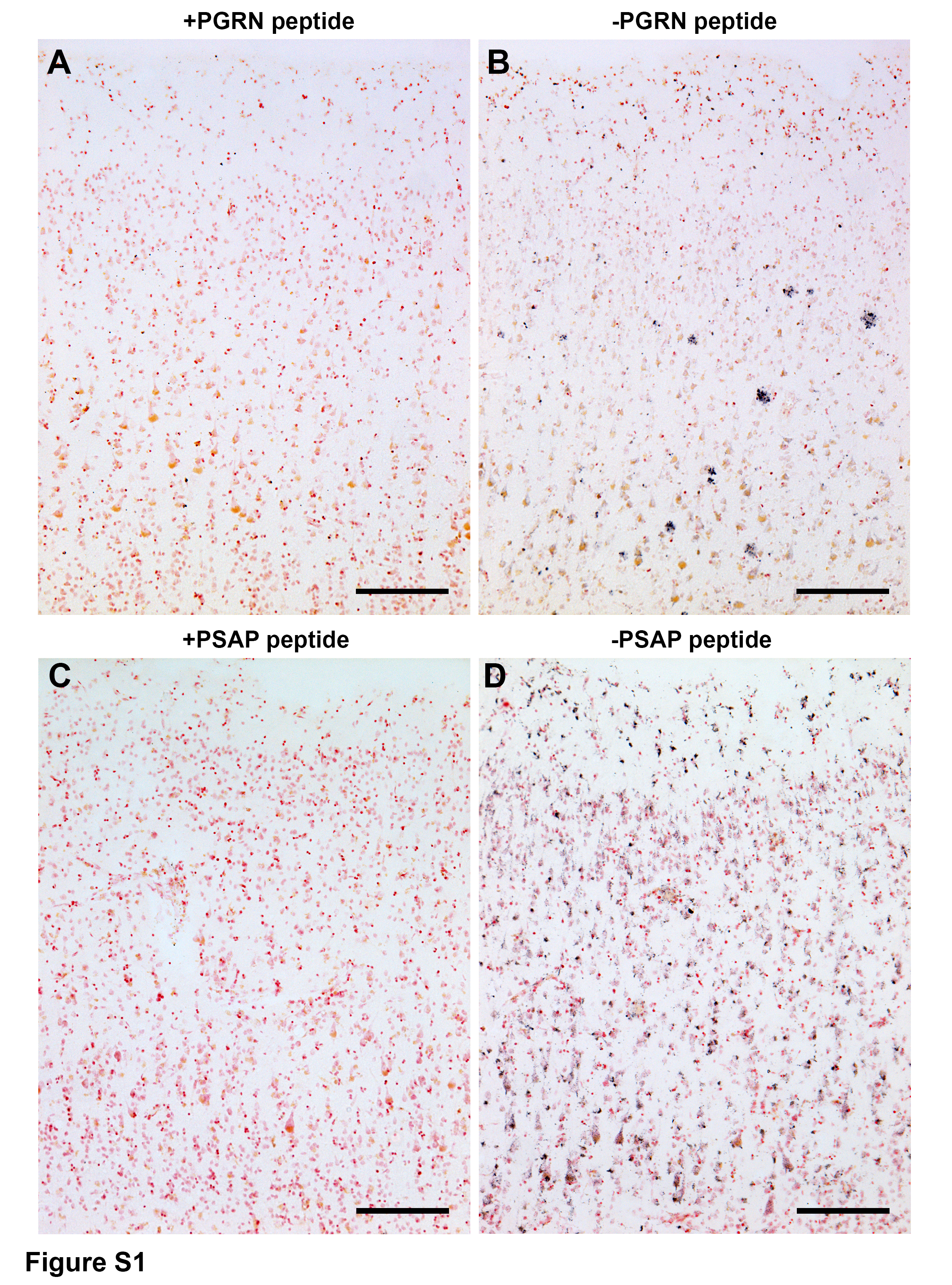

Supplement: Supplementary file 1 — Additional file 1 Figure S1. (A-B). PGRN peptide absorption of goat anti-PGRN antibody (AF2420). Low magnification images demonstrating absence of immunoreactive structures in MTG (AD case) reacted with PGRN antibody preabsorbed with PGRN peptide (A)(+PGRN peptide) and demonstration of significant immunoreactivity in parallel section from same case reacted with non-peptide antibody (B)(−PGRN peptide). Scale bars represent 200 μm. (C-D). PSAP peptide absorption of rabbit anti-PSAP antibody (AF8470). Low magnification images demonstrating absence of immunoreactive structures in MTG (AD case) reacted with PSAP antibody preabsorbed with PSAP peptide (C)(+PSAP peptide) and demonstration of significant immunoreactivity in parallel section from same case reacted with non-peptide antibody (D)(−PSAP peptide). Scale bars represent 200 μm. [file 40478_2019_862_MOESM1_ESM.tif]

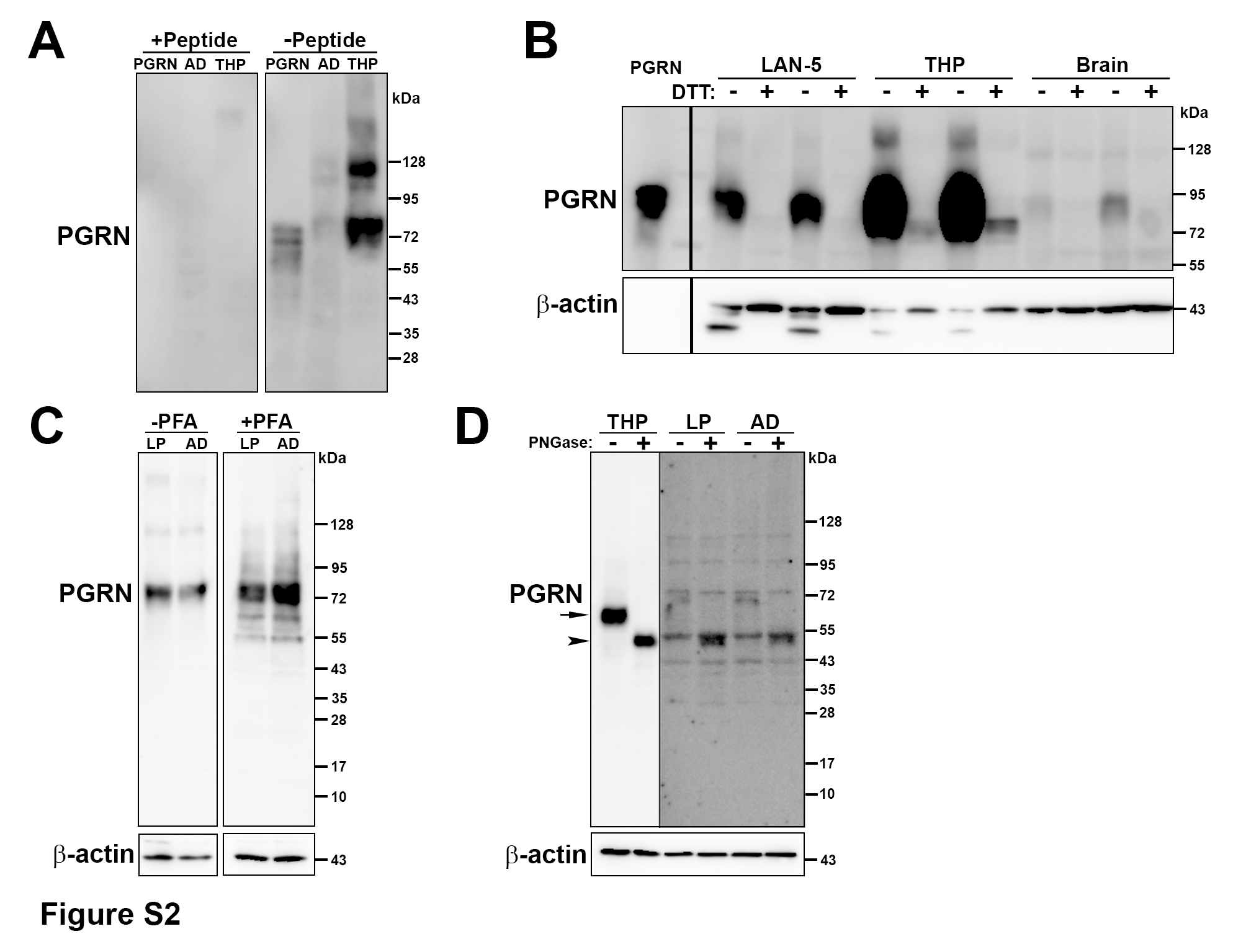

Supplement: Supplementary file 2 — Additional file 2 Figure S2. Western blot characterization of PGRN polypeptides detected with goat anti-PGRN antibody (AF2420). A). Absorption of PGRN polypeptides with peptide-absorbed PGRN antibody. Western blot images of purified PGRN peptide (PGRN), AD brain protein extract (AD) and THP macrophage cell protein extracts (THP) detected with PGRN peptide-absorbed antibody (+Peptide) or non-absorbed antibody (−Peptide). Peptide absorption resulted in almost complete absence of PGRN polypeptide bands. B). Detection of PGRN by western blots is sensitive to reducing agents. Western blots comparing polypeptide bands in protein extracts of samples prepared with DTT (+) or without DTT (−) as reducing agent. Samples: PGRN; purified recombinant PGRN protein. LAN-5; neuronal cells. THP; THP-1 derived macrophages. Brain; AD brain samples. Blots were probed with goat anti-PGRN antibody (R&D Systems, AF2420:50 ng/ml). C). Sensitivity of detection of PGRN polypeptides in brain samples is enhanced by membrane fixation with paraformaldehyde vapors. Western blot images of brain protein samples from low plaque (LP) and Alzheimer’s disease (AD) cases separated under identical conditions without reducing agents. One membrane was fixed with paraformaldehyde vapors (+PFA) compared to membrane not PFA treated (−PFA). Sensitivity of detection is enhanced in PFA fixed membranes. Blots were probed with goat anti-PGRN antibody (R&D Systems, AF2420:50 ng/ml). D). Identification of deglycosylated forms of PGRN. Protein extracts from THP macrophage cells (THP), and LP and AD brain samples were treated with deglycosylation enzyme PNGaseF (+) or control treated (−). Deglycosylation treatment resulted in increased levels of 55 kDa polypeptides and reduced amounts of ~ 75 kDa PGRN band. Blots were probed with goat anti-PGRN antibody (R&D Systems, AF2420:50 ng/ml). [file 40478_2019_862_MOESM2_ESM.tif]

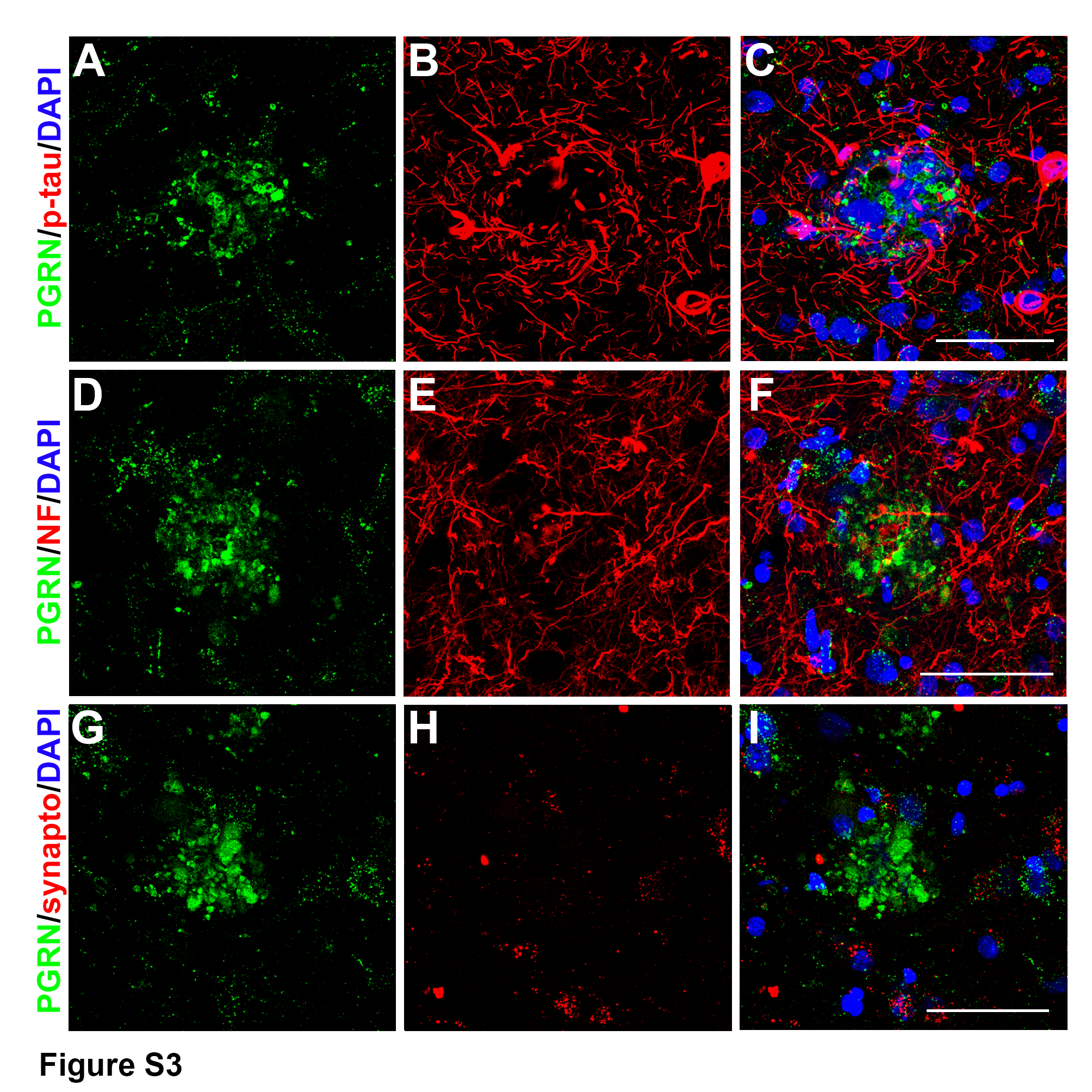

Supplement: Supplementary file 3 — Additional file 3 Figure S3 Limited colocalization of PGRN with markers defining neurites and neuritic plaques. A-C): Confocal image of PGRN (green)(A) immunoreactivity associated with a neuritic plaque identified with pTau antibody AT180 (B) with merged image (C) in an AD case showing no colocalization of staining. DAPI staining identified nuclei and also highly aggregated amyloid within plaques. Scale bar represents 20 μm. D-F): Confocal image of PGRN (green)(D) immunoreactivity associated with neurites and neuritic plaque identified with pan-neurofilament antibody SMI312 (E) with merged DAPI-stained image (F) in an AD case showing limited colocalization of staining. Scale bar represents 20 μm. G-I): Confocal image of PGRN (green)(G) immunoreactivity associated with neurites and neuritic plaque identified with synaptophysin antibody (H) with merged DAPI-stained image (I) in an AD case showing no colocalization of staining. Scale bar represents 20 μm. These images were acquired using a Leica SP8 confocal microscope. [file 40478_2019_862_MOESM3_ESM.tif]

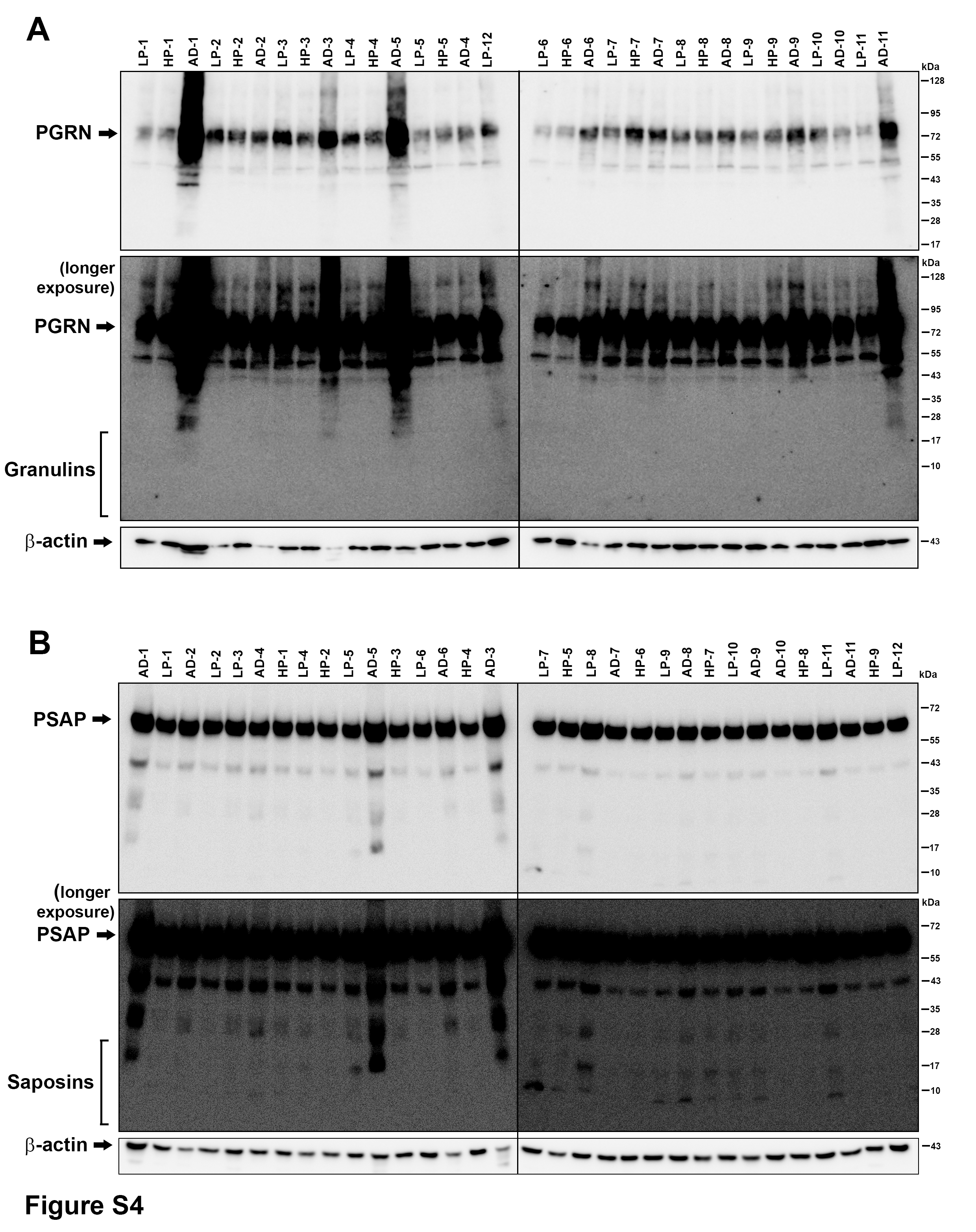

Supplement: Supplementary file 4 — Additional file 4 Figure S4. Complete western blot images of MTG protein samples. A). Complete western blot images of MTG brain sauremples probed with antibody to detect PGRN polypeptides in Low plaque (LP), high plaque (HP) and AD samples. Major PGRN polypeptide bands detected at approximately 75–80 kDa, with less intense bands at 55 kDa. These blots showed absence of low molecular weight granulin peptides. Top Image: Shorter exposure of western blot. Lower Image: Longer exposure of western blot to demonstrate if granulin peptides were present. B). Complete western blot images of MTG brain samples probed with antibody to detect PSAP polypeptides in Low plaque (LP), high plaque (HP) and AD samples. Major PSAP polypeptide bands detected at approximately 72 kDa. Additional polypeptide bands are detectable with image enhancement indicating detection of PSAP-derived saposin peptides. Top Image: Shorter exposure of western blot. Lower image: Longer exposure of western blot images of MTG brain samples probed with antibody to detect PSAP polypeptides. Bands with molecular weights of saposin peptides are indicated. These were noticeable in AD samples. [file 40478_2019_862_MOESM4_ESM.tif]

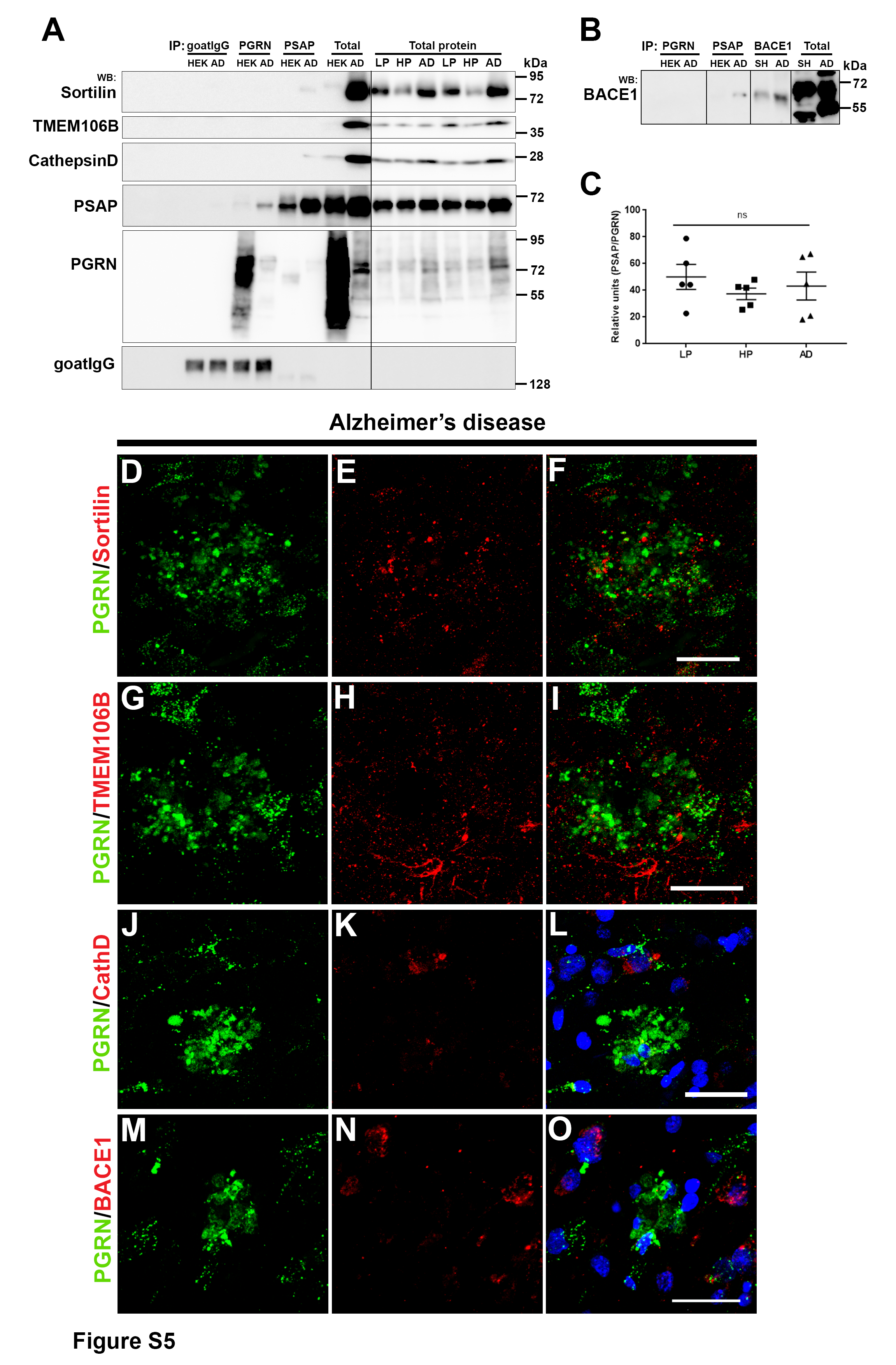

Supplement: Supplementary file 5 — Additional file 5 Figure S5. Additional analysis and controls for proteins analyzed by co-immunoprecipitation or colocalization with progranulin and prosaposin A-B) Composite western blot of additional potential PGRN-interacting proteins using Protein A or Protein G beads conjugated with normal goat IgG, or antibodies to PGRN, PSAP, sortilin, TMEM106B, cathepsin D and beta secretase-1 (BACE-1) (panel B). Comparison was carried out with non-immunoprecipitated total protein extracts of brain samples analyzed on the same blots. Sortilin: Antibody detected polypeptide with expected molecular weight in brain samples. No sortilin immunoreactive bands were present in PGRN IP samples but a faint band was detected in PSAP IP samples. TMEM106B: Antibody detected polypeptide with expected molecular weight in brain samples. No TMEM106B immunoreactive bands were present in PGRN or PSAP immunoprecipitated samples. Cathepsin D: Antibody detected polypeptide with expected molecular weight in brain samples. No cathepsin D immunoreactive bands were present in PGRN IP samples but a faint band was detected in PSAP IP samples. BACE1: Analysis showed that samples precipitated with PGRN conjugated beads did not pull down BACE1 immunoreactive bands but band detected in PSAP-immunoprecipitated samples. BACE-1 conjugated beads included as positive control. (SH – extract of neuronal SH-SY5Y cells). C) Scatter blot showing ratio of expression levels of PSAP to amounts of PGRN in immunoprecipitated samples. The ratio of PSAP to PGRN present in samples immunoprecipitated with antibody to PGRN was not significantly different between disease groups. Confocal microscopy of PGRN-positive plaque structures with indicated antibodies. D-F) PGRN (green) and Sortilin (red) immunoreactivity was detected in plaques but there was no detectable colocalization (F). Scale bars represent 30 μm. G-I) PGRN (green) and TMEM106B (red) immunoreactivity was detected in or around plaques but there was no detectable co [file 40478_2019_862_MOESM5_ESM.tif]

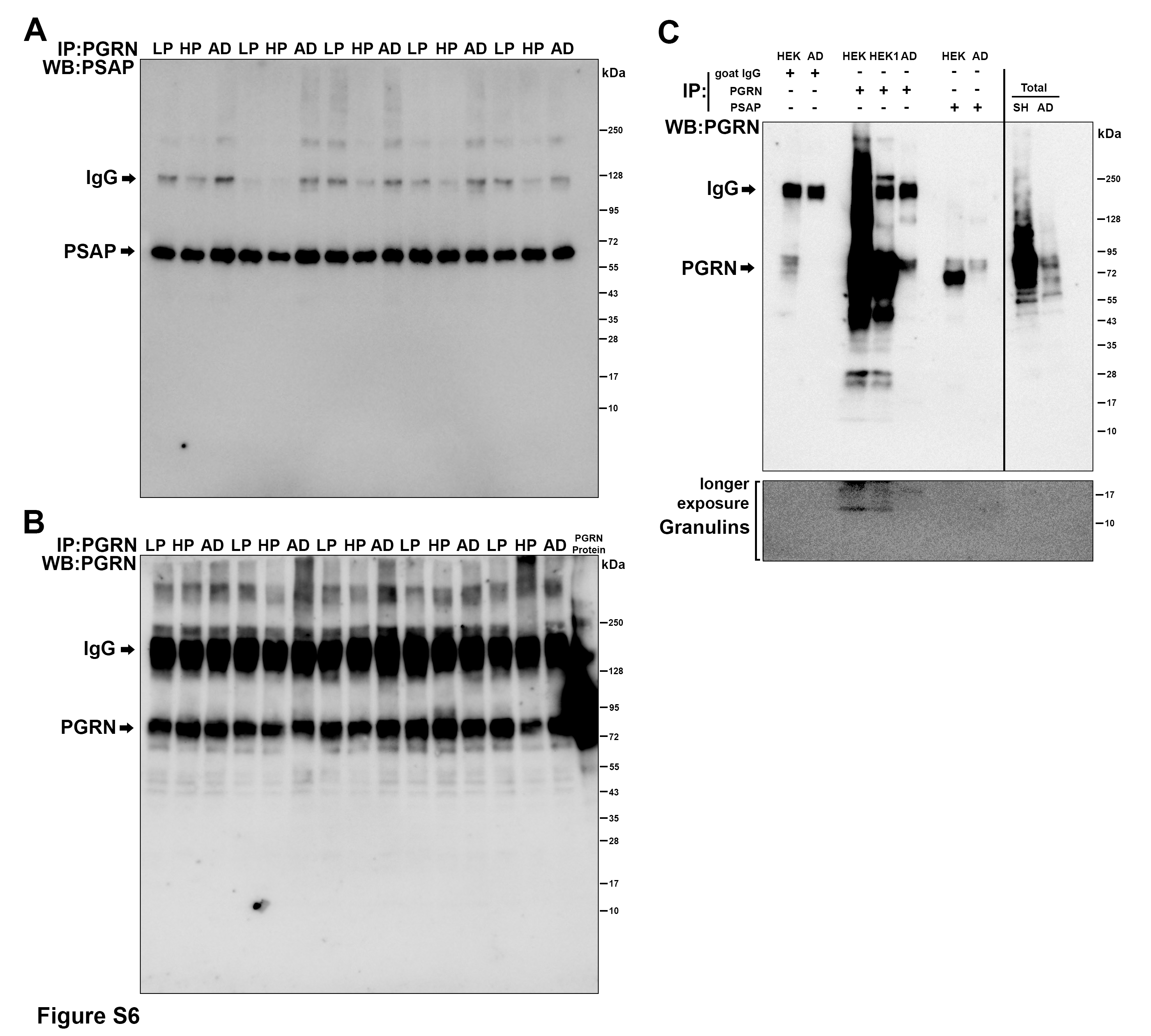

Supplement: Supplementary file 6 — Additional file 6 Figure S6 Complete western blot images of immunoprecipitated brain and cell samples to identify presence of granulin or saposin peptides. A) Complete western blot image of brain samples immunoprecipitated with PGRN antibody conjugated magnetic beads and detected with antibody to PSAP. The blots showed no evidence of lower molecular weight saposin peptides. B) Complete western blot image of brain samples immunoprecipitated with PGRN antibody conjugated magnetic beads and detected with antibody to PGRN. The blots showed no evidence of lower molecular weight granulin peptides. C) Complete western blot images of PGRN-overexpressing HEK and brain samples (AD) immunoprecipitated with magnetic beads conjugated with control normal goat immunoglobulin (goat IgG), or PGRN or PSAP antibodies and probed with antibody to PGRN. Image shows that granulin peptides could be minimally detected only in HEK cells expressing high levels of PGRN protein. Blot also shows total extracts from SH-SY5Y differentiated neurons (SY) and AD brain analyzed in parallel showing no detectable granulin peptides. [file 40478_2019_862_MOESM6_ESM.tif]

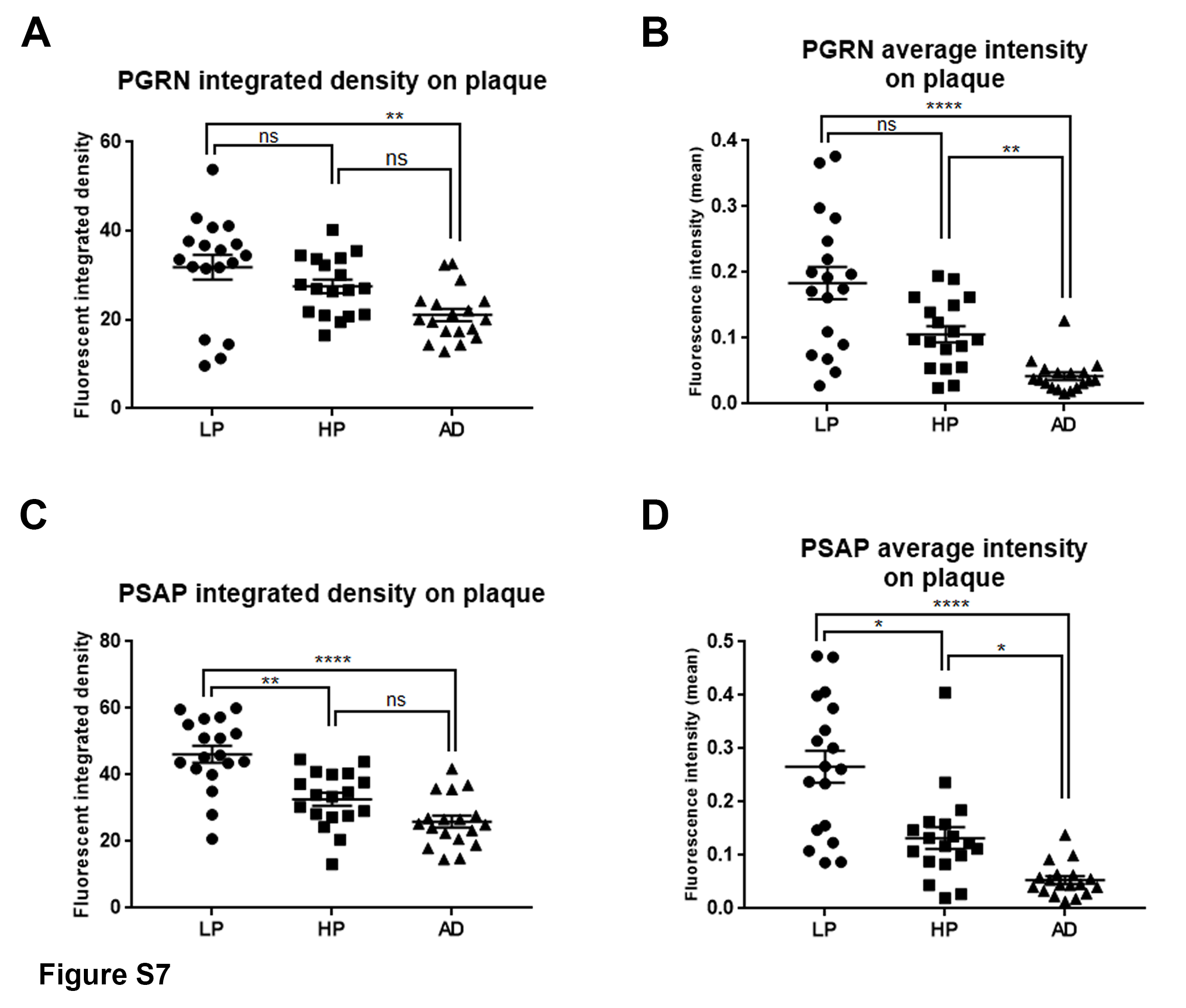

Supplement: Supplementary file 7 — Additional file 7 Figure S7: Fluorescent plaque intensities of PGRN and PSAP in MTG sections between disease groups. A) Fluorescent intensity of PGRN immunoreactivity on plaques between LP, HP and AD cases. Six plaques were measured in 3 cases in each group (n = 18 plaques/group). Results represent mean + SEM of total fluorescent intensity. Results analyzed by one-way ANOVA with Neuman-Keuls post-hoc test between groups. ** p < 0.01, ns: not significant. B) Area-adjusted fluorescent intensity of PGRN immunoreactivity on plaques between LP, HP and AD cases of PGRN-PSAP positive plaques. Six plaques were measured in 3 cases in each group (n = 18 plaques/group). Results represent mean ± S.E.M. of average fluorescent intensity adjusted for plaque area (Fig. 9N). Results analyzed by one-way ANOVA with Neuman-Keuls post hoc test between groups. **** p < 0.0001, ** p < 0.01, ns: not significant. C) Fluorescent intensity of PSAP immunoreactivity on plaques between LP, HP and AD cases. Six plaques were measured in 3 cases in each group (n = 18 plaques/group). Results represent mean ±S.E.M. of total fluorescent intensity. Results analyzed by one-way ANOVA with Neuman-Keuls post hoc test between groups. **** p < 0.0001, ns: not significant. D) Area-adjusted fluorescent intensity of PSAP immunoreactivity on plaques between LP, HP and AD cases of PGRN-PSAP positive plaques. Results represent mean + ±S.E.M. of average fluorescent intensity adjusted for plaque area measures (Fig. 9N). Six plaques were measured in 3 cases in each group (n = 18 plaques/group). Results analyzed by one-way ANOVA with Neuman-Keuls post-hoc test between groups. **** p < 0.0001, * p < 0.05, ns: not significant. [file 40478_2019_862_MOESM7_ESM.tif]
